# Tumor-Induced Osteomalacia: A Systematic Clinical Review of 895 Cases

**DOI:** 10.1007/s00223-022-01005-8

**Published:** 2022-07-20

**Authors:** Ariadne Bosman, Andrea Palermo, Julien Vanderhulst, Suzanne M. Jan De Beur, Seiji Fukumoto, Salvatore Minisola, Weibo Xia, Jean-Jacques Body, M. Carola Zillikens

**Affiliations:** 1 Department of Internal Medicine, Erasmus MC, University Medical Center Rotterdam, Rotterdam, The Netherlands; 2 Unit of Metabolic Bone and Thyroid Disorders, Fondazione Policlinico Universitario Campus Bio-Medico, Rome, Italy; 3 Department of Medicine, CHU Brugmann, Université Libre de Bruxelles (ULB), Brussels, Belgium; 4 Johns Hopkins University School of Medicine, Baltimore, MD USA; 5 Fujii Memorial Institute of Medical Sciences, Institute of Advanced Medical Sciences, Tokushima University, Tokushima, Japan; 6 Department of Clinical, Internal, Anesthesiological and Cardiological Sciences, “Sapienza” Rome University, 00161 Rome, Italy; 7 Department of Endocrinology, Key Laboratory of Endocrinology, The National Commission of Health, Peking Union Medical College Hospital, Chinese Academy of Medical Sciences, Beijing, China

**Keywords:** Tumor-induced osteomalacia, FGF23, Osteomalacia, Rickets, Hypophosphatemia, Fracture

## Abstract

Tumor-induced osteomalacia (TIO) is a rare and largely underdiagnosed paraneoplastic condition. Previous reviews often reported incomplete data on clinical aspects, diagnosis or prognosis. The aim of this study was to present a systematic clinical review of all published cases of TIO. A search was conducted in Pubmed, Embase, Web of Science from inception until April 23rd, 2020. We selected case reports and case series of patients diagnosed with TIO, with information on tumor localization and serum phosphate concentration. Two reviewers independently extracted data on biochemical and clinical characteristics including bone involvement, tumor localization and treatment. 468 articles with 895 unique TIO cases were included. Median age was 46 years (range 9 months–90 years) and 58.3% were males. Hypophosphatemia and inappropriately low or normal 1,25-dihydroxyvitamin D levels, characteristic for TIO, were present in 98% of cases. Median tumor size was 2.7 cm (range 0.5 to 25.0 cm). Serum fibroblast growth factor 23 was related to tumor size (*r* = 0.344, *P* < 0.001). In 32% of the cases the tumor was detected by physical examination. Data on bone phenotype confirmed skeletal involvement: 62% of cases with BMD data had a T-score of the lumbar spine ≤ − 2.5 (*n* = 61/99) and a fracture was reported in at least 39% of all cases (*n* = 346/895). Diagnostic delay was longer than 2 years in more than 80% of cases. 10% were reported to be malignant at histology. In conclusion, TIO is a debilitating disease characterized by a long diagnostic delay leading to metabolic disturbances and skeletal impairment. Increasing awareness of TIO should decrease its diagnostic delay and the clinical consequences.

**Supplementary Information:**

The online version contains supplementary material available at 10.1007/s00223-022-01005-8.

## Introduction

The rare and debilitating condition of tumor-induced osteomalacia (TIO), also known as oncogenic or oncogenous osteomalacia, is nowadays more frequently recognized, especially since its pathophysiological mechanisms are better understood. In this paraneoplastic disease, the tumor secretes phosphaturic factors known as “phosphatonins” [1–3], amongst which fibroblast growth factor 23 (FGF23) is the most frequently found, leading to the cardinal features of the disease: hypophosphatemia from renal phosphate wasting, reduced 1,25-dihydroxyvitamin D concentration through inhibition of its synthesis, rickets in children and osteomalacia in adults, with diffuse bone pain, fractures and muscle weakness. Radical resection of the responsible tumor leads to a rapid normalization of biochemical parameters and to marked improvement or resolution of the symptoms [4]. The majority of the tumors are benign and arise from mesenchymal tissue [5].

Since the initial presentation can be misleading or non-specific, TIO still remains a diagnostic and therapeutic challenge despite the progress made in its understanding. Moreover, the lack of serum phosphate measurement in many standard comprehensive chemistry panels contributes to its delayed diagnosis [6]. In addition, the causative tumor can develop anywhere in the body and can be small enough to elude even our modern imaging techniques [7, 8]. The consequence can be a long diagnostic delay, leading sometimes to a dramatic outcome with multiple fractures and severe disability. In order to identify tumor mass, a stepwise imaging approach using functional and anatomic imaging is suggested. Several techniques are being used to detect the tumor, including but not limited to computed tomography (CT) or magnetic resonance (MR), 18F-fluorodeoxyglucose (FDG) PET/CT, Technetium 99 m octreotide, scintigraphy/SPECT/CT and Gallium-68 (^68^ Ga)-DOTATATE PET/CT. Recent studies have demonstrated that 68 Ga-DOTATATE PET/CT shows the greatest accuracy in TIO localization [6, 9, 10].

A few clinical reviews aimed to investigate the clinical profile of TIO but they presented significant limitations: the authors included cases of acquired hypophosphatemic rickets / osteomalacia even if the tumor was not found [4, 7, 11–14]. The overall aim of this review is to carefully describe the clinical and biochemical aspects and the bone phenotype of TIO by conducting a complete analysis of all published cases between 1947 and 2020. Although in clinical practice the diagnosis often can be suspected based on the clinical characteristics and the biochemical findings, we chose a more precise approach. To avoid uncertain or incorrect diagnoses, the current study focuses only on cases where the causative tumor was localized and treatment led to cure or marked improvement of the patient’s condition. While performing our review, another systematic review was published on patients with a clinical diagnosis of TIO [15]. Our approach enabled us to describe more precisely the clinical presentation and the localization of the responsible tumor in patients with TIO.

## Methods

### Data Sources and Searches

This review was conducted following the Preferred Reporting Items for Systematic Reviews and Meta-analyses statement (PRISMA) [16]. Due to the nature of our research question and inclusion criteria, we did not perform a separate risk of bias assessment. We searched Pubmed, Embase and Web of Science from inception to April 23rd 2020, without language restrictions. We screened references lists of included articles. Search terms focused on tumor-induced osteomalacia and hypophosphatemic rickets (Online Appendix Table 1).

### Study Selection

Two reviewers screened all articles and abstracts of conferences containing descriptions of clinical cases of TIO. The criteria appraised to select cases were as follows: acquired hypophosphatemia due to renal phosphate wasting with a reported serum phosphate level before treatment, no known family history of osteomalacia, reported localization of the causative tumor, and cure after appropriate treatment or at least clear-cut improvement (in terms of clinical and biochemical parameters or in the amount of medical treatment needed). Case reports on linear sebaceous naevi [17, 18], von Recklinghausen disease [19, 20], fibrous dysplasia of bone [21], McCune-Albright syndrome [22], and hematological malignancies [23–25] were excluded. These conditions are known to be potentially associated with hypophosphatemia but are better described as “tumor-induced osteomalacia like syndrome” [14]. In addition, some cases were published more than once (80 reports) and only the first publication was included.

### Data Extraction

Two investigators reviewed all cases. When available, the following information was collected: demographic data (age, sex); data about the disease (time from the first symptoms to diagnosis and treatment, outcome after treatment, duration of follow-up); data about the tumor (location, signs at physical examination pointing out to the tumor, presence or absence of symptoms that could be attributed to the tumor directly, techniques used for localization, histology, malignant features and size of the tumor); biochemical data (serum phosphate, 25-hydroxyvitamin D, 1,25-dihydroxyvitamin D concentration, plasma FGF23 concentration and tubular maximal phosphate resorption/glomerular filtration rate (TmP/GFR)) and data about bone health (occurrence of fractures and bone mineral density (BMD) at the lumbar spine, total hip and femoral neck assessed by dual-energy X-ray absorptiometry). TmP/GFR, if not directly available in the manuscript, was calculated using the nomogram of Walton & Bijvoet [26]. FGF23 values were recorded, taking into account the assay used and the normal range. Since FGF23 was measured with several different commercial or in-house kits and since they are not interconvertible [27], we expressed FGF23 values as the times of the upper limit of the respective normal ranges.

Serum phosphate and TmP/GFR were expressed in mmol/L and 1,25-dihydroxyvitamin D concentrations were expressed in pmol/L with the conversion factors of 0.323 and 2.4 to convert from mg/dL and pg/mL, respectively. 25-hydroxyvitamin D concentrations were expressed in ng/mL.

A tumor was considered malignant when either mentioned as such or when an invasive behavior was evident, such as the occurrence of metastases.

### Statistical Analysis

Data are presented as mean ± SD or median with the interquartile range according to distribution. Statistical tests used for comparisons were the Mann Whitney-U test, Kruskal–Wallis test and chi-square test for homogeneity. Spearman correlation analysis was used to analyze the correlations among continuous variables. All analyses were performed with IBM SPSS software, version 25 (SPSS, Chicago, IL).

## Results

**Fig. 1:**
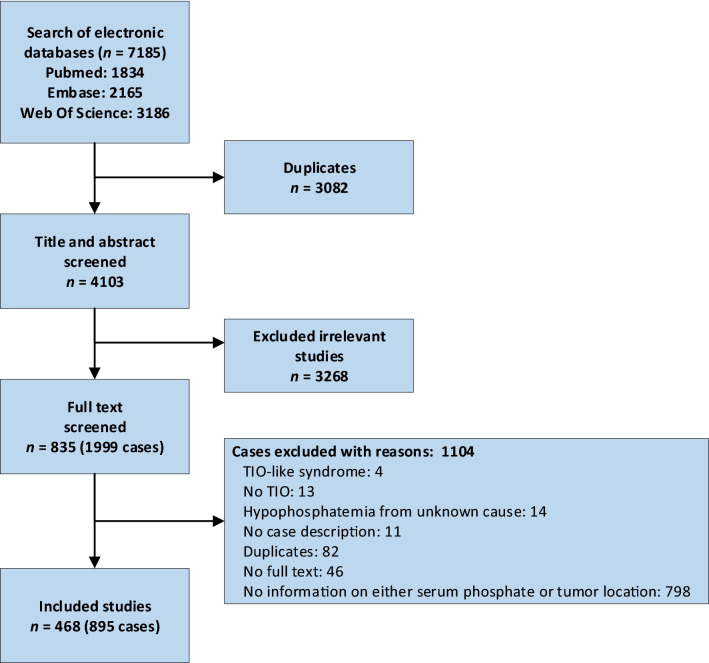
Flow diagram of the search

We identified 468 articles on 895 unique cases of TIO, spanning a period from 1947 until 2020 (Fig. 1), and they are reported in Online Resource 1. Less than 5% of cases were published before 1980 and more than 70% were published in the last decade, suggesting that the disease becomes more frequently recognized with time (Online Appendix Fig. 1).

### Demographic Data

The disease occurred at any age from 9 months to 90 years with a median age at the time of diagnosis of 46 years. The majority of patients were aged between 30 and 60 years with a peak between 45 and 55 years of age (Online Appendix Fig. 2). Of the 858 cases with information on sex, 41.7% were females and 58.3% were males.

### Biochemical Data

In adults, mean serum phosphate was 0.48 ± 0.15 mmol/L (*N* = 829; normal range 0.74–1.52) [28] and 96.1% had hypophosphatemia. The median TmP/GFR was 0.36 mmol/L (from 0.02 to 1.80; *N* = 357; normal range 0.81–1.36) [26]. In patients under 18 years old, these values were 0.59 ± 0.26 mmol/L (*N* = 38) and 0.37 mmol/L (from 0.14 to 0.92; *N* = 14), respectively.

**Fig. 2:**
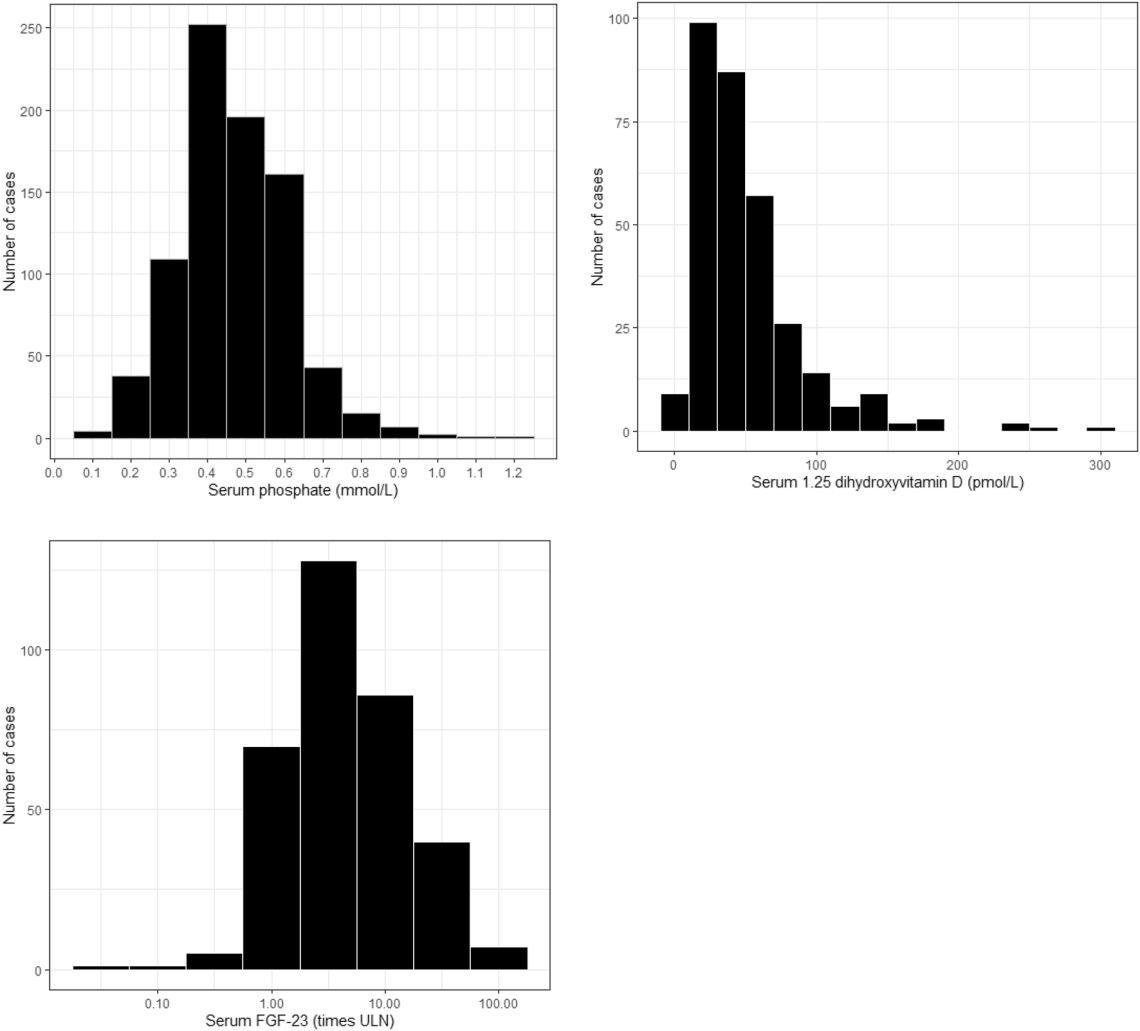
Distribution of serum phosphate, 1.25 (OH)2 D levels and the times of the upper limit of normal FGF-23 among adults with TIO. Histograms showing the distribution of serum phosphate in mmol/L (**A**), serum 1.25(OH)2 Vitamine D in pmol/L (**B**) and the times of the upper limit of normal of FGF-23 (**C**). Normal range for serum phosphate: 0.74–1.52 mmol/L. Normal range for serum 1.25(OH)2 Vitamin D: 50–155 pmol/L. *FGF-23* Fibroblast growth factor 23, *ULN* upper limit of normal

One of the cardinal features in TIO is a low or inappropriately normal circulating concentration of 1,25-dihydroxyvitamin D since hypophosphatemia is expected to stimulate renal 1α-hydroxylase which will increase 1,25-dihydroxyvitamin D production. The median value of 1,25-dihydroxyvitamin D concentration was 51.1 pmol/L (from undetectable to 301.6 pmol/L; *N* = 337; normal range: 50–155) (Fig. 2). More than 60% of the patients had 1,25-dihydroxyvitamin D values below the lower limit of the normal range. About one third of the values lied within the normal range and 8 patients had elevated values of 1,25-dihydroxyvitamin D, of whom 5 patients had elevated values of parathyroid hormone with low or normal calcium levels. Thus, more than 97% of 1,25-dihydroxyvitamin D values were low or inappropriately normal. There was a significant positive correlation between 1,25-dihydroxyvitamin D and serum phosphate level (*r* = 0.227; *P* < 0.001; *N* = 337). The median value of 25-hydroxyvitamin D concentration was 23.5 ng/mL (from undetectable to 150.0 ng/mL; *N* = 373; normal range 25–80). The median FGF23 value was 3.75 times the upper limit of the normal range (xULN) (0.0–162; *N* = 346). Over 80% of the results lied between more than 1 to 21 times xULN (Fig. 2). In 31 of 346 cases, FGF23 was below the upper limit of normal, varying from 0.02 to 0.99 times the upper limit of normal. Four of these cases had a tumor size smaller than 1.5 cm, eight cases had a tumor size 1.5–3.0 cm. We found a positive correlation between FGF23 (xULN) and tumor size (*r* = 0.344, *P* < 0.001; *N* = 130) (Online Appendix Fig. 3). There was a significant negative correlation between FGF23 and serum phosphate (*r* = − 0.114 *P* = 0.034; *N* = 346) and between FGF23 and Tmp/GFR (*r* = − 0.243; *P* = 0.001; *N* = 187). The correlation between FGF23 and serum 1,25-dihydroxyvitamin D was not significant (*P* = 0.443). FGF-23 levels were not always reported. Therefore, we analyzed the differences between cases who had FGF-23 measured with cases for which no FGF23 levels were reported. Interestingly, tumor size was significantly smaller in patients who had FGF23 measurements (median tumor size 2.5 cm) than in patients without reported FGF23 measurements (median tumor size 2.9 cm; *P* = 0.013). Moreover, TmP/GFR was slightly lower in patients without FGF23 measurements (*P* = 0.010). The diagnostic delay seemed slightly shorter in the cases with FGF23 measurements, but this was not significant (Online Appendix Table 2).

### Tumor Characteristics

**Fig. 3:**
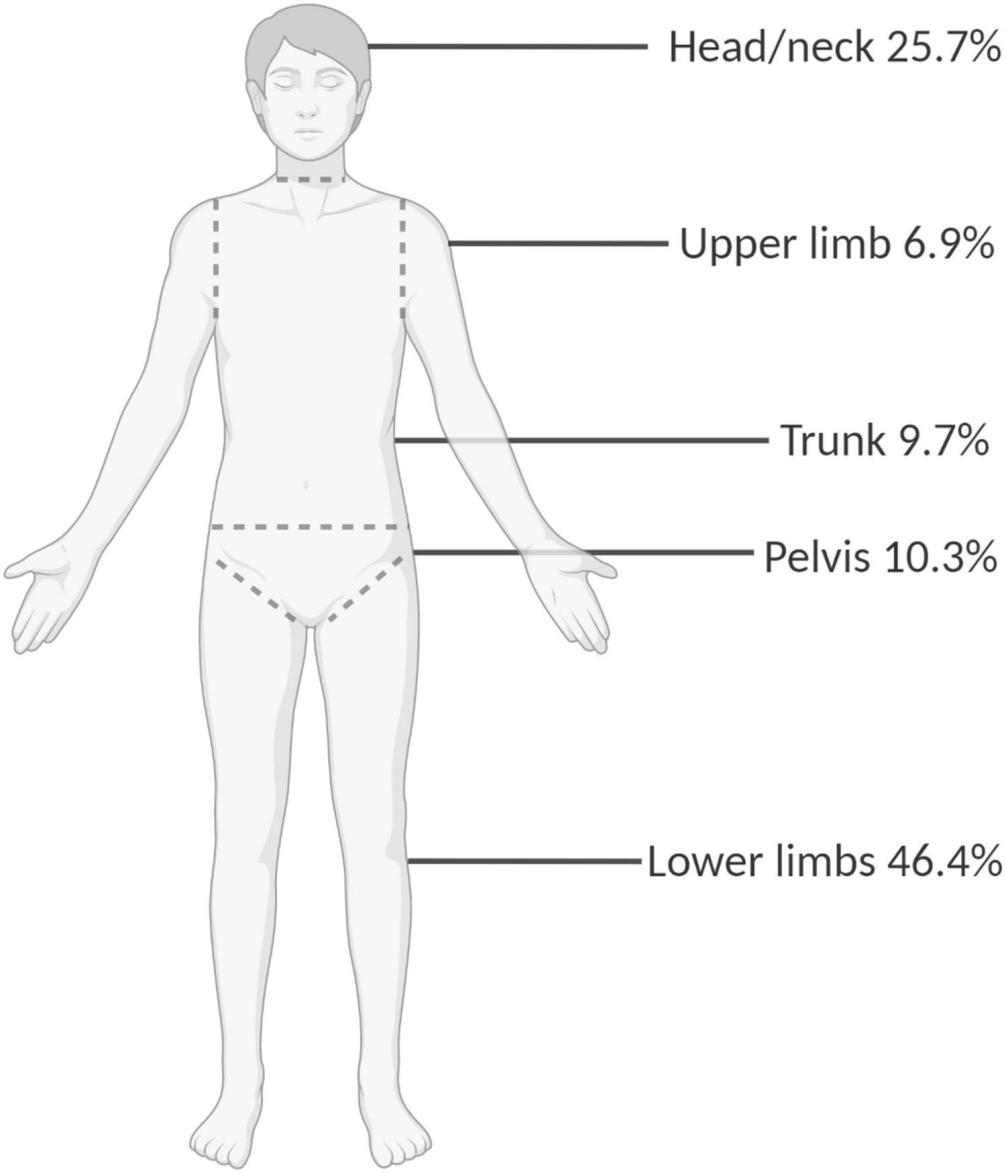
Localization of the tumor

We considered five regions: head and neck, trunk, pelvis, upper and lower limbs. The two most frequent localizations were the lower limbs (46.4%) and the head and neck area (25.7%) (Fig. 3). The median size of the tumor was 2.7 cm ranging from 0.5 to 25 cm (*N* = 416). No relationship was found between tumor size and serum phosphate levels (*P* = 0.12), TmP/GFR (*P* = 0.63) or 1,25-dihydroxyvitamin D levels (*P* = 0.44). However, a weak but positive correlation was found between the size of the tumor and diagnostic delay (*r* = 0.113, *P* = 0.033; *N* = 354). Table 1 depicts the differences between tumor sizes ranging from tumors smaller than 1.5 cm to larger than 5.0 cm. The diagnostic delay and FGF23 levels were significantly different between the different tumor sizes.

**Table 1:** Differences between tumors of different sizes in adults

	Tumor < 1.5 cm		Tumor 1.5–3 cm		Tumor 3–5 cm		Tumor > 5 cm		*P* value*
	*N*		*N*		*N*		*N*		
Age, years	63	46.0 (21.0, 73.0)	151	46.0 (18.0, 76.0	125	46.0 (19.0, 73.0)	54	46.0 (18.0, 90.0)	0.999
Diagnostic delay, years	53	3.0 (0.25, 19.0)	128	3.0 (0.5, 20.0)	109	3.0 (0.17, 27.0)	48	5.5 (0.17, 21.0)	0.032
Phosphate, mmol/L	63	0.48 (0.23, 0.87)	151	0.48 (0.19, 0.90)	125	0.45 (0.11, 1.03)	54	0.42 (0.16, 0.90)	0.320
TmP/GFR, mmol/L	31	0.36 (0.09, 1.30)	72	0.39 (0.02, 1.25)	52	0.36 (0.09, 1.50)	17	0.31 (0.11, 0.74)	0.768
Calcium, mmol/L	49	2.25 (1.10, 2.86)	106	2.28 (2.00, 2.64))	88	2.26 (1.98, 10.50)	37	2.30 (1.95, 2.70)	0.146
FGF23 xULN	24	2.53 (0.56, 15.56)	57	2.78 (0.13, 48.67)	35	5.06 (0.28, 139.19)	12	4.66 (1.03, 45.00)	0.005
BMD T-score L1-L4	13	− 2.7 (− 4.5, − 1.3)	36	− 3.1 (− 5.9, 0.1)	12	− 2.2 (− 4.8, − 1.1)	10	− 2.8 (− 6.9, − 1.2))	0.861
BMD T-score Total hip	3	− 2.6 (− 3.9, − 2.5)	14	− 2.5 (− 5.0, − 0.8)	1	−	3	− 2.9 (− 5.9, − 1.2)	0.936
BMD T-score femoral neck	6	− 3.4 (− 5.3, − 0.8)	19	− 2.8 (− 4.5, 0.4)	6	− 2.8 (− 4.6, − 2.2)	5	− 3.0 (− 3.6, − 1.5)	0.749
Tumor localization	63		151		125		54		0.073
Lower limb		41 (61.5%)		78 (51.7%)		55 (44.0%)		25 (46.3%)	
Upper limb		2 (3.2%)		7 (4.6%)		12 (9.6%)		6 (11.1%)	
Head/neck		10 (15.9%)		39 (25.8%)		30 (24.0%)		9 (16.7%)	
Trunk		6 (9.5%)		10 (6.6%)		12 (9.6%)		10 (5.6%)	
Pelvis		4 (6.3%)		17 (11.3%)		15 (12.0%)		3 (5.6%)	
Multiple locations		0 (0.0%)		0 (0.0%)		1 (0.8%)		1 (1.9%)	

*Differences between groups were tested using Mann Whitney-U test, Kruskal–Wallis test and chi-square test for homogeneity. Continous data are presented as median (range). Categorical data are presented as count (%)

*BMD* bone mineral density, *FGF23* Fibroblast growth factor 23, *TmP/GFR* maximum tubular reabsorption rate of phosphate, *xULN* times the upper limit of normal

Since these tumors can be found anywhere in the body, we determined how frequently they could be detected readily on just clinical examination. From the total of 895 descriptions, 494 cases reported a physical examination. 160 tumors, i.e., 32.4% of the cases with reported physical examination and 17.9% of the total number of cases, were reported to have been identified at physical examination. For tumors larger than 5.0 cm, physical examination identified the tumor in 59.5% of the cases with a reported physical examination (*N* = 25/42) and 42.4% of the total number of cases (*N* = 25/59). In the 461 adult cases with physical examination, external tumors were slightly larger than tumors that were not identified at physical examination, with a median size of 3.0 cm (range 0.5–15.0 cm) vs. 2.5 cm (0.6–15.0 cm), respectively (*P* < 0.001) (Table 2). The time to diagnosis was not significantly different between external tumors and tumors that were not identified by physical examination: 4.0 years (0.2–25 years) vs. 3.0 years (0.1–42 years), respectively (*P* = 0.06). Online Appendix Fig.  4 depicts the identification of external and internal tumors by year of publication.

**Table 2:** Differences between external and internal* tumors in adults

	External		Internal*		*P* value^†^
	*N*		*N*		
Age, years	154	44.5 (18.0, 79.0)	307	47.0 (18.0, 90.0)	0.057
Diagnostic delay, years	141	4.0 (0.2, 25.0)	235	3.0 (0.1, 42.0)	0.055
Tumor size, cm	105	3.0 (0.5, 15.0)	150	2.5 (0.6, 15.0)	< 0.001
Phosphate, mmol/L	154	0.47 (0.11, 1.20)	307	0.45 (0.10, 0.90)	0.991
Calcium, mmol/L	115	2.30 (1.95, 2.77)	211	2.25 (1.27, 2.90)	0.070
TmP/GFR, mmol/L	62	0.26 (0.03, 0.74)	130	0.36 (0.02, 1.80)	0.004
FGF23 ULN	30	3.8 (0.3, 63.0)	115	3.7 (0.3, 62.6)	0.938
BMD T-score L1-L4	19	− 3.5 (− 5.5, − 0.5)	38	− 2.7 (− 5.9, 3.7)	0.106
BMD T-score total hip	6	− 2.7 (− 5.9, − 0.9)	14	− 3.3 (− 5.0, − 0.2)	0.444
BMD T-score femoral neck	11	− 3.6 (− 7.4, − 1.7)	23	− 2.5 (− 5.3, 1.6)	0.019
Tumor localization	154		307		
Lower limb		108 (35.2%)		72 (46.8%)	< 0.001
Upper limb		13 (4.2%)		16 (10.4%)	
Head/neck		88 (28.7%)		44 (28.6%)	
Trunk		49 (16.0%)		13 (8.4%)	
Pelvis		45 (14.7%)		8 (5.2%)	
Multiple locations		4 (1.3%)		1 (0.6%)	

*Internal tumors were defined as tumors that were not identified at physical examination. ^†^ Differences between groups were tested using Mann Whitney-U test, Kruskal–Wallis test and chi-square test for homogeneity. Continuous data are presented as median (range). Categorical data are presented as count (%)

*BMD* bone mineral density, *FGF23* Fibroblast growth factor 23, *TmP/GFR* maximum tubular reabsorption rate of phosphate, *ULN* upper limit of normal

In this review, 56 tumors out of 579 (9.7%) were reported to be malignant at histology. The size of the tumor tended to be larger in the group with a malignant tumor: 5.3 cm (1.2–15.0 cm) vs. 2.5 cm (0.5–15 cm) (*P* < 0.001) (Online Appendix Table 3). Smaller tumors (i.e., less than 3 cm) were almost always benign (97.9%). Malignant tumors were most frequently diagnosed as PMT (phosphaturic mesenchymal tumor) (19.6%), followed by osteosarcomas (7.1%) while amongst PMTs, 2.4% were found to be malignant.

Tumors responsible for TIO are very heterogeneous. However, since the work of Weidner et al. [5] and Folpe et al. [29] who also reviewed previous cases, it appears that the majority was considered to be a PMT (65.4%; *N* = 527/806), followed by hemangiopericytoma (9.1%; *N* = 81/806), giant cell tumor (2.9%; *N* = 26/806) and hemangioma (2.1%; *N* = 19/806). If one considers only the cases published after the publication by Folpe et al. in 2004, PMT account for 75.5% (*N* = 512/678) of the cases. The second most frequent histological diagnoses was hemangiopericytoma (6.3%; *N* = 48/678), followed by giant cell tumor (2.6%; *N* = 20/678).

### Clinical Characteristics

Apart from the symptoms secondary to osteomalacia, such as proximal muscle weakness or pain, a tumor can lead by its anatomical location to specific symptoms such as tenderness, paresthesias, paresis anosmia, nasal obstruction or epistaxis. Out of 895 cases, 74 experienced local symptoms indicating that a thorough medical history and physical examination can give a clue as to the tumor localization in at least 8.3% of the cases. Interestingly, in 42 out of 216 cases (19.4%) with a tumor in the head and neck region, local symptoms were described that might in theory have led to tumor localization on clinical grounds. In 32.4% of cases where a physical examination was performed, a tumor was identified (*N* = 160/494). Out of the 334 cases were a tumor could not be identified on physical examination, 9.3% experienced local symptoms such as redness, swelling, pain. Taken together, 21.3% (*n* = 191/895) of the tumors in the total population or 38.7% (*N* = 191/494) of the cases with a reported physical examination could have been localized just on clinical grounds.

Symptoms secondary to osteomalacia, such as proximal muscle weakness or pain, occurred in at least 89.9% of cases and seemed more prevalent in cases with tumors located in the upper and lower limb and head and neck region (91.9%, 91.3% and 90.9%, respectively) than in the trunk and pelvis (82.8% and 85.9%, respectively). However, the occurrence of symptoms secondary to osteomalacia was not significantly different between localizations of the tumor (*P* = 0.108).

We next evaluated how many cases described the occurrence of fractures. 422 out of 895 cases evaluated and described whether a fracture occurred. Strikingly, 346 of these patients (82.0% of 422 and 38.7% of the total) had one or more recent fractures (exact frequency not always described). 41.0% (*N* = 142/346) suffered one fracture, 24.5% (*N* = 85/346) suffered two fractures, 25.4% (*N* = 88/346) suffered three fractures and 9.0% (*N* = 31/346) suffered four or more fractures. The majority of these patients had a hip fracture (56.6%, *N* = 196), followed by a rib fracture (51.4%, *N* = 178) and a vertebral fracture (38.4%, *N* = 133).

TIO is by definition a defect in bone mineralization. In addition, we were interested in the effect of the disease on bone density. The lumbar spine BMD was described in only 95 adult cases (11.4%). Strikingly, 57 out of these 99 cases (60.0%) had a T-score below -2.5. The BMD of the femoral neck was analyzed only in 47 cases (5.7%), but also here 32 cases (68.1%) had a T-score below -2.5. No correlation was found between lumbar spine BMD and serum phosphate (*N* = 95), FGF23 (*N* = 47) or tumor size (*N* = 71).

We next evaluated which imaging techniques were used to detect the tumors. Most tumors were located using MRI (36.8%), followed by CT scan (29.3%), ^18^F-fluoro-deoxy-glucose (FDG)-PET/CT (15.8%) and X-ray (10.3%). ^68^Gallium-DOTATATE, -DOTANOC and -DOTATOC PET/CT scanning together located 20.3% of the tumors. Other less commonly used techniques were nuclear imaging, ultrasound and angiography.

The time gap from the initial patient’s complaints to the diagnosis and cure was highly variable, ranging from 0.1 to 42 years with a median delay of 3.5 years. Only 20% of case were correctly diagnosed within 2 years while in 30% it took between 5 and 25 years to diagnose TIO (Online Appendix Fig.  5). In addition, physicians did not perform better during the last decade despite the availability of modern imaging armamentarium. Before 2010, the median delay was 3.5 years, after 2010, the median delay was still 3.4 years (*P* = 0.608).

We found a positive correlation between tumor size and the diagnostic delay (*r* = 113, *P* = 0.033, *N* = 354). In addition, Table 2 shows that both diagnostic delay and FGF23 levels increase with tumor size. Specifically, tumors > 5 cm had a median diagnostic delay of 5.5 years, while tumors 1.5–3.0 cm had a median diagnostic delay of 3.0 years (*P* = 0.015).

### Treatment

The administration of phosphate supplementation to treat the hypophosphatemia was reported in 461/895 cases. 81.6% (*N* = 376/461) of these cases were treated with phosphate supplementation. Calcitriol or alphacalcidol was administered in 76.9% (*N* = 332/432) of cases with information on calcitriol use. Apart from phosphate and active vitamin D, most tumors were treated with surgery only (84.2%, *N* = 754/829) or with a combination of surgery and chemotherapy or radiotherapy (4.2%). Chemotherapy, radiotherapy and radiofrequency ablation as a single treatment entity were used in a few cases (*N* = 27). In all cases, there was an improvement or a normalization of the biochemical parameters. Malignant tumors were managed with surgery only (50.0%), chemotherapy and/or radiotherapy in addition to surgery (32.0%), chemotherapy only (14.0%) or radiotherapy only (2.0%).

A follow-up duration of more than 6 months was reported in 325 cases. The duration of follow-up in this group varied from 0.6 to 26 years with a median of 2.0 years. Recurrence was reported in 16.1% (*N* = 50/310) among which 20.0% were malignant and 80.0% were benign. As expected, the proportion of local recurrence was significantly higher in malignant tumors (36.4% vs. 15.8%; *P* = 0.034; *N* = 224). Out of the 754 surgically treated cases, 279 had a minimal follow-up duration of 6 months. 14.2% of these patients (*N* = 38/267) reported recurrence of disease during follow-up.

## Discussion

In this review, we describe the clinical and biochemical aspects and the bone phenotype of published TIO cases. Several reviews about TIO have been published previously [4, 7, 11, 13, 14, 30, 31], but they provide incomplete clinical details because they did not include a systematic review of all the published cases of TIO until now. Recently, Rendina et al. performed a systematic review on TIO, but they also included cases with only a clinical diagnosis of TIO [15]. In the current study, we included cases with a reported serum phosphate before treatment, a localized tumor and cure after appropriate treatment or at least marked improvement, thereby excluding cases with uncertain or incorrect diagnoses. Careful inspection of each published case—including also non-English publications –, allowed us to better characterize the clinical features of this condition and to draw conclusions on tumor size, diagnostic delay and bone phenotype hitherto not reported.

Our data show that the majority of TIO occurs in adults, mainly in their forties and fifties. However, also cases have been described in very young children implicating that if in childhood inherited conditions cannot be demonstrated, TIO should be suspected and looked for. We found that TIO was more prevalent in males with 58% of cases, which is in agreement with the recent systematic review performed by Rendina et al., but in contrast with previous literature [4, 7, 15].

The diagnosis of TIO depends on clinical evaluation, biochemical testing and tumor identification [32]. According to our review, a thorough clinical examination can result in identification of the tumor. Patients were often aware of a lump or a growth for many years and in some cases the tumors caused symptoms other than those related to osteomalacia, mostly due to the compression of adjacent vascular or neurological structures. In sum, in nearly 40% of the patients with reports on clinical evaluation, the tumor could have been localized just on clinical grounds. This is quite a high percentage and has not been reported before, but the literature on this topic is scarce. Shah et al. reported on 163 previously published cases of TIO in the head and neck region in whom the tumor was localized using clinical evaluation in 16.7% of cases (*N* = 22/131)[33]. Similarly, we found that 19.4% of cases with a tumor in the head and neck region reported local symptoms. It should be noted that we do not have information on the timing of the tumor detection during clinical examination. Our review only includes cases with a localized tumor and it is possible that identification of the tumor on imaging aided in linking local symptoms to the tumor location.

It has been well known that bone involvement represents one of the most important metabolic consequences of TIO. Transiliac bone biopsy samples from TIO subjects showed a severe condition of osteomalacia with low mineralized bone volume, low mineralized trabecular thickness, and a significant increase in the heterogeneity of mineralization [34, 35]. We found that 60.0% of cases with a report on BMD showed a low bone mass (based on T-scores ≤ − 2.5). The tremendously high rate of fractures in our study population (82% reported one or more fractures) confirms how bone fragility represents a hallmark of the disease. This is in line with previous reports on the severe decrease of BMD at any investigated skeletal sites [36, 37] and impairment of bone microarchitecture and strength [38] with consequent fragility fractures which frequently occur in weight-bearing bones [36, 39]. The fracture rate reported in our study is higher than the fracture rate reported by Rendina et al. (56% in men and 48% in women), which may be explained by the fact that we applied different inclusion criteria or because we distinguish between articles that reported on fracture occurrence and articles that did not. Nevertheless, our findings imply that the bone phenotype is a hallmark of the disease and confirm the need for bone evaluation in patients with chronic hypophosphatemia [40].

As to the location of the tumors, they were found anywhere in the body but most often in the lower limbs (46.4%) and in the head and neck area (25.7%). This is in line with the publication by Rendina et al., who reported that 56% of the tumors in men and 49% of the tumors in women were located in the lower extremities and 27% of the tumors in men and 34% of the tumors in women were located in the head and neck region [4, 7]. These two sites should thus be explored with scrutiny if the tumor is not readily found.

The size of the tumors varied considerably, from 0.5 to 25 cm, but the largest ones (> 5 cm) were found predominantly in the lower limbs. Our analyses showed that tumor size did not correlate with serum phosphate, TmP/GFR or 1,25-dihydroxyvitamin D but it was significantly correlated with FGF23 levels. This is in line with previous literature and might suggest that in an untreated patient, an increase in FGF23 level over time could represent an increase in tumor size [41]. This finding underlines the importance of timely tumor localization and removal.

The diagnostic delay for TIO has been reported to range from 2.5 to 28 years [32]. Similarly, we found a median time gap between the initial presentation and the tumor-related treatment of 3.5 years with a range from 0.1 to 25 years. Surprisingly, this delay has not fallen significantly in the last decade despite the availability of very powerful imaging techniques, suggesting that the awareness of the condition is still a major determinant in the celerity of the diagnosis. This is supported by the finding that the diagnostic delay was not significantly different for external tumors compared to internal tumors. Interestingly, tumors larger than 5 cm had a median diagnostic delay of 5.5 years, while tumors less than 5 cm had a median diagnostic delay of 3.0 years. In addition, tumors were larger in the group without FGF23 measurements. Possible explanations for the longer delay in the group with the largest tumor are: the lack of FGF23 measurements, and the lack of experience of the treating physician in recognizing this condition.

Hypophosphatemia and renal phosphate wasting are cardinal features in TIO. However, since serum phosphate is not always included in the standard chemistry panel, failure to identify or recognize low serum phosphate levels can delay the diagnosis. For this reason, we make the case that serum phosphate should always be measured in case of muscle weakness or pain, bone pain and fatigue, with or without fractures. Measurement of serum FGF23 is relevant in any patient with hypophosphatemia and renal phosphate wasting [32], and in the current review it was never found below the limit of the normal range. However, in a few cases FGF23 was within the normal range, which is still abnormal in the setting of hypophosphatemia [42]. A possible explanation for this is the role of other inhibitors of phosphate transport such as Fibroblast growth factor 7 or secreted frizzled-related protein 4 [43, 44]. Measurement of intact FGF23 seems to be the most specific and sensitive test in TIO [45]. However, raised FGF23 cannot definitively establish the diagnosis of TIO since there is a considerable overlap in FGF-23 levels between TIO and inherited conditions of hypophosphataemia [42], TIO-like syndromes [46], recent renal transplantation [47], and drug-related hypophosphatemia, mainly due to intravenous iron [48–50] Still, FGF23 levels are generally higher in TIO than in XLH and other causes of hypophosphatemia [42]. Rendina et al. found that serum levels of intact FGF23 were higher in patients with a localized tumor compared to patients in whom the tumor was not identified, which raises the question whether these patients were correctly diagnosed as TIO.

Histology has been simplified since the work of Weidner et al. [5] in 1987 and Folpe et al. [29] in 2004. Prior to these studies, many tumors were qualified as e.g., hemangiopericytomas, hemangiomas, giant cell tumors and osteoblastomas. Both Weidner et al. and Folpe et al. reviewed the histology of tumors that were involved in TIO and concluded that many of them could be reclassified as PMT, a morphologically distinct entity that can be further classified into four groups: mixed connective tissue, osteoblastoma-like, non-ossifying fibroma-like and ossifying fibroma-like. Nevertheless, not all causative tumors fall in this group, as revised diagnoses still included other tumors e.g., hemangiopericytomas and hemangiomas [5, 29]. In this review, we found that the majority of causative tumors was classified as PMT (65.4%), followed by hemangiopericytoma and giant cell tumor. When we considered only the cases that were published after the publication by Folpe et al. in 2004 [29], PMT account for 75.5% of the cases. These results demonstrate that the classification of the tumors has improved and that the histopathologic characteristics of PMT are recognized. Nevertheless, the histological classification of PMT does not entirely correlate with the clinical presentation, since not all PMTs lead to hypophosphatemia [51].

In this review, most of the tumors were benign. It’s important to underline that about 10% of cases were reported to be malignant at histology. This percentage is higher than reported by Rendina et al., but not all TIO cases included in our review reported information on histology. Amongst PMTs, we found that only 2.4% was malignant. Also non-PMT malignant tumors can secrete FGF23 leading to hypophosphatemia with renal phosphate wasting [52, 53]. This may worsen the burden of symptoms associated with the cancer and may warrant additional treatment with phosphate supplements and active vitamin D metabolites.

The only curative treatment for TIO is removal of the tumor. Hence, localization of the tumor is very important. Often a multimodality approach is necessary, including functional imaging followed by anatomical imaging of suspicious areas [32, 54]. Due to the nature of this study we cannot draw conclusions on the best performing imaging techniques in TIO. It is likely that negative results of imaging modalities were not reported, which precludes us from making statements about the sensitivity and specificity of the different imaging techniques that are available. Our data suggest that in the last two decades more internal tumors were found, implying that localization techniques have become more efficient. However, despite the availability of advanced imaging techniques, we did not see a decrease in diagnostic delay in the last ten years, indicating that recognition of the clinical characteristics of TIO needs to be improved.

As to the treatment, complete surgical resection with wide margins when feasible is the optimal treatment [55]. Incomplete resection is usually not sufficient for full resolution of the symptoms and can lead to recurrence. In those cases, phosphate and active metabolite of vitamin D supplements can be continued [14]. We found that the majority of patients were treated with surgery only (84.2%) or with a combination of surgery and chemotherapy or radiotherapy (4.2%). However, it should be noted that we only included cases in whom the tumor was localized and tumor-related treatment led to marked improvement or cure. Cases in whom a tumor was identified but tumor-related treatment was not initiated, not possible or did not result in improvement in clinical and biochemical parameters, were not included. Therefore, we cannot draw any conclusions on treatment effectiveness. Furthermore, we found that 81.6% of cases were treated with phosphate supplementation and 76.9% of cases were treated with calcitriol before any tumor-related treatment, which shows that the importance of medical therapy in TIO before tumor removal is well recognized.

Lastly, we found a recurrence rate of 14.2% in surgically treated patients. This is higher than what was recently reported by Li. et al.[55], who observed a recurrence rate of 7.8% after primary surgery in 230 retrospectively analysed patients from a single tertiary hospital in China. Nevertheless, although many cases did not report on follow-up duration or recurrence, these results suggest that surgical treatment for TIO is highly effective and should be considered in all patients with localized tumors.

Our review has a few weaknesses. We have only included cases with a reported localized tumor and we excluded cases where the tumor could not be located despite exhaustive investigation [56–58]. It is likely that these cases do not get published, which may have resulted in an overrepresentation of the less challenging cases in our review. Second, the variables assessed in this study are extracted from the case reports. For this reason, there is a considerable amount of missing data. Moreover, there is a variability related to the different assays of FGF23 used in terms of sensitivity and/or specificity, and unknown timing of the measurements and/or influence of medical therapy, which could have concealed or decreased correlations for example between FGF23 and phosphate and tumor size. Third, the concept of disease duration may be elusive as symptoms such as weakness are difficult to date. Fourth, most of the case reports reported that subjects were not taking calcitriol when they performed 1,25 (OH)2D measurements; however, few authors did not include this information in the publication.

In conclusion, our review on all TIO cases published until now aims to help clinicians to define the clinical, biochemical and radiological profile of this rare condition. By including only cases with a localized tumor and serum phosphate report, we were able to draw conclusions on the significance of clinical examination, bone evaluation and tumor size, hitherto not reported. From our findings we can conclude that there is still a considerable diagnostic delay, despite increased knowledge of TIO and improved imaging techniques, resulting in metabolic disturbances and skeletal impairment. A thorough clinical examination can point to the causative tumor. Moreover, we found that FGF23 was related to tumor size, a finding that underlines the importance of early detection of the causative tumor followed by its removal.

## Supplementary Information

Below is the link to the electronic supplementary material.

Supplementary file1 (DOCX 129 kb)

Supplementary file2 (DOCX 66 kb)
