# Supplementary material for: Tumor-Induced Osteomalacia: A Systematic Clinical Review of 895 Cases
Source: Calcif Tissue Int. 2022 Jul 20;111(4):367–79. doi: 10.1007/s00223-022-01005-8 (PMC9474374; doi:10.1007/s00223-022-01005-8)

**Tumor-induced osteomalacia: a systematic clinical review of 895 cases**

**Authors:** Ariadne Bosman^1^*, Andrea Palermo^2^*, Julien Vanderhulst^3^, Suzanne Jan De Beur^4^ , Seiji Fukumoto^5^, Salvatore Minisola^6^, Weibo Xia^7^, Jean-Jacques Body^3^, M. Carola Zillikens^1^.

*These authors contributed equally to this work

**Author Affiliations:**

^1^ Erasmus MC, University Medical Center Rotterdam, The Netherlands, Department of Internal Medicine.

^2^ Unit of Metabolic bone and thyroid disorders, Fondazione Policlinico Universitario Campus Bio-Medico, Rome, Italy

^3^ Department of Medicine, CHU Brugmann, Université Libre de Bruxelles (ULB), Brussels, Belgium.

^4^ Johns Hopkins University School of Medicine, Baltimore, MD, USA

^5^ Fujii Memorial Institute of Medical Sciences, Institute of Advanced Medical Sciences, Tokushima University, Tokushima, Japan

^6^ Department of Clinical, Internal, Anesthesiological and Cardiological Sciences, "Sapienza" Rome University, 00161 Rome, Italy

^7^ Department of Endocrinology, Key Laboratory of Endocrinology, The National Commission of Health, Peking Union Medical College Hospital, Chinese Academy of Medical Sciences, Beijing, China

**Corresponding author:**

M. Carola Zillikens

Erasmus MC, University Medical Center Rotterdam, The Netherlands, Department of Internal Medicine

Email: m.c.zillikens@erasmusmc.nl

**APPENDIX MATERIAL**

**Appendix Table 1.** Search Strategies

| **Source** | **Search** |
| --- | --- |
| Pubmed | (“Oncogenic osteomalacia”[tiab] OR “oncogenous osteomalacia”[tiab] OR “Rickets, Hypophosphatemic” [MESH] OR “hypophosphatemic rickets”[tiab] OR “tumor-induced osteomalacia”[tiab] OR “tumor-induced rickets”[tiab] OR “oncogenic rickets”[tiab]) |
| Embase | ('oncogenic osteomalacia'/exp OR 'oncogenic osteomalacia':ab,ti OR 'oncogenous osteomalacia':ab,ti OR 'hypophosphatemic rickets':ab,ti OR 'tumor-induced osteomalacia':ab,ti OR 'tumor-induced rickets':ab,ti OR 'oncogenic rickets':ab,ti) |
| Web of Science | (TOPIC: ("oncogenic osteomalacia" OR "oncogenous osteomalacia" OR "hypophosphatemic rickets" OR "tumor-induced osteomalacia" OR "tumor-induced rickets" OR "oncogenic rickets") |

**Appendix Figure 1.** Year of publication


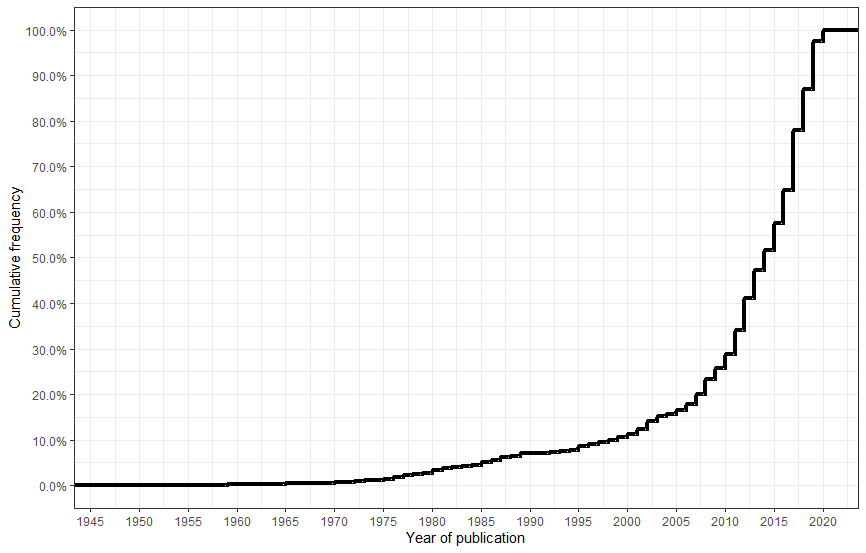


**Appendix Figure 2.** Distribution of age at diagnosis


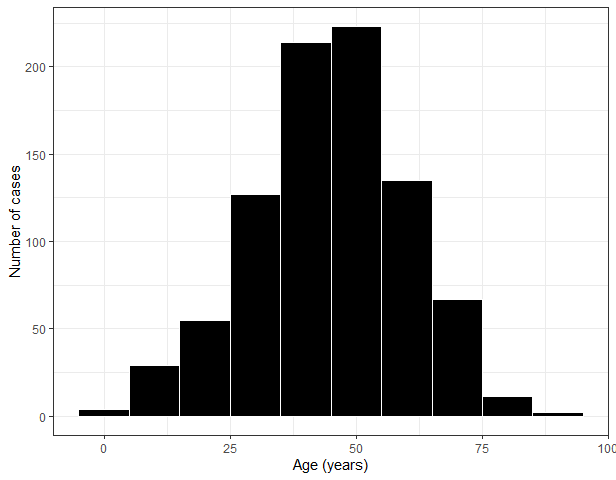


**Appendix Figure 3**. Scatterplot of tumor size plotted against xULN FGF-23


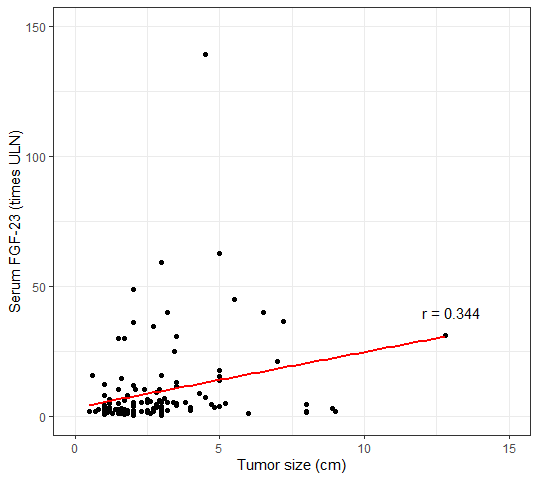


**Appendix Table 2.** Differences between adult patients with and without FGF-23 measurements

|  | FGF23 measured | | FGF23 not measured | |  |
| --- | --- | --- | --- | --- | --- |
|  | N | Median (range) | N | Median (range) | P value |
| Age, years | 355 | **48.0 (18, 90)** | **477** | **45.0 (18, 88)** | **0.011** |
| Diagnostic delay, years | 254 | 3.0 (0.08, 42.0) | 363 | 4.0 (0.17, 38.0) | 0.218 |
| **Tumor size, cm** | **128** | **2.5 (0.6, 12.8)** | **265** | **2.9 (0.5, 15.0)** | **0.013** |
| Phosphate, mmol/L | 355 | 0.48 (0.10, 1.07) | 474 | 0.45 (0.11, 1.20) | 0.259 |
| TmP/GFR, mmol/L | 185 | **0.39 (0.02, 1.50)** | **172** | **0.32 (0.03, 1.80)** | **0.010** |

Data presented as median (range). Abbreviations: FGF23, Fibroblast Growth Factor 23; TmP/GFR, maximum tubular reabsorption rate of phosphate.

**Appendix Figure 4.** Identification of external vs internal tumors by year of publication


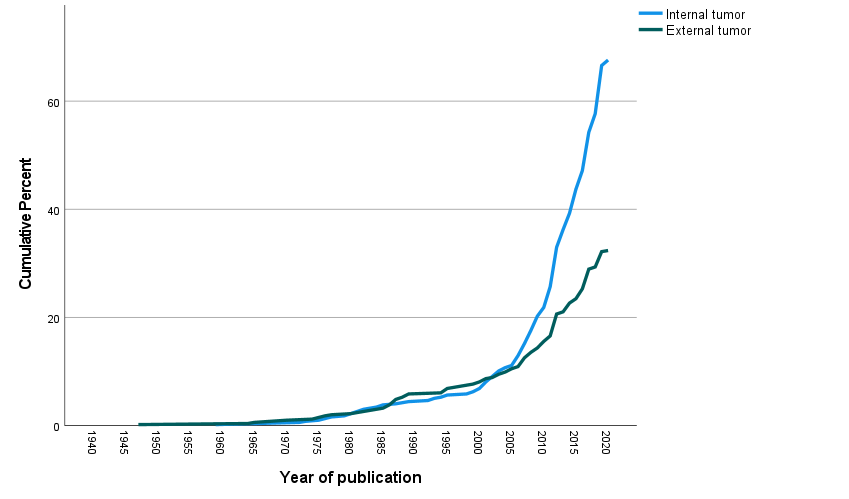


**Appendix Table 3.** Differences between malignant and benign isolated tumors in adult patients

|  | Malignant | | Benign | |  |
| --- | --- | --- | --- | --- | --- |
|  | N | Median (Range) | N | Median (Range) | P value |
| Age, years | 56 | 55.0 (18.0, 88.0) | 523 | 46.0 (18.0, 90.0) | 0.003 |
| Diagnostic delay, years | 39 | 3.0 (0.12, 19.0) | 393 | 4.0 (0.1, 38.0) | 0.086 |
| Tumor size, cm | 18 | 5.3 (1.2, 15.0) | 257 | 2.5 (0.5, 15.0) | <0.001 |
| Phosphate, mmol/L | 56 | 0.39 (0.13, 1.20) | 523 | 0.45 (0.10, 1.07) | 0.002 |
| Calcium, mmol/L | 40 | 2.24 (1.27, 2.90) | 363 | 2.25 (1.10, 10.50) | 0.527 |
| TmP/GFR, mmol/L | 20 | 0.26 (0.04, 1.80) | 242 | 0.36 (0.02, 1.60) | 0.223 |
| FGF23 times ULN | 21 | 4.8 (1.2, 32.0) | 223 | 3.7 (0.02, 139.2) | 0.299 |
| BMD T-score L1-L4 | 5 | -2.4 (-3.5, -1.2) | 70 | -3.1 (-6.9, 0.1) | 0.174 |
| BMD T-score total hip | 2 | -1.3 (-1.4, -1.2) | 24 | -2.95 (-4.0, -0.9) | 0.074 |
| BMD T-score femoral neck | 1 | - | 31 | -3.2 (-7.4, -1.5) | 0.278 |

Data presented as median (range). Abbreviations: BMD, bone mineral density; FGF23: Fibroblast growth factor 23; TmP/GFR, maximum tubular reabsorption rate of phosphate; ULN, upper limit of normal.

**Appendix Figure 5.** Cumulative frequency of the diagnostic delay overall and during the last 10 years, calculated as the time gap between clinical presentation and diagnosis, in years


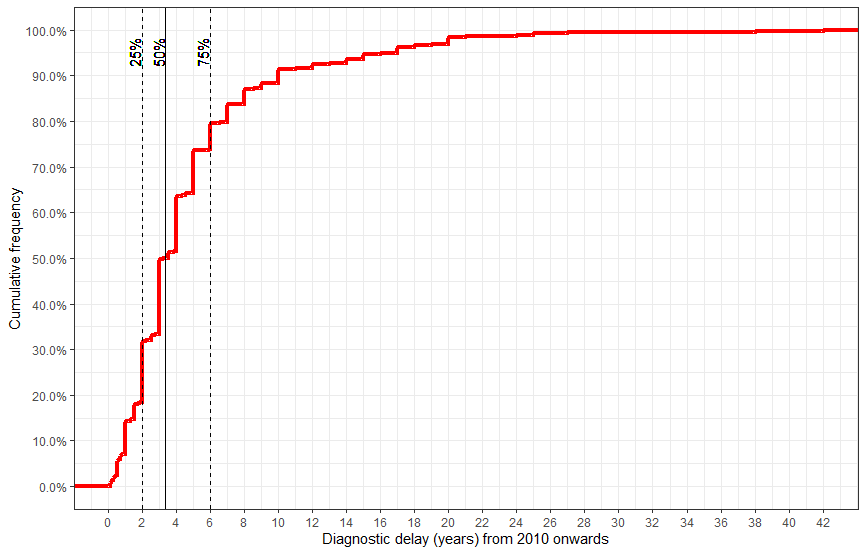

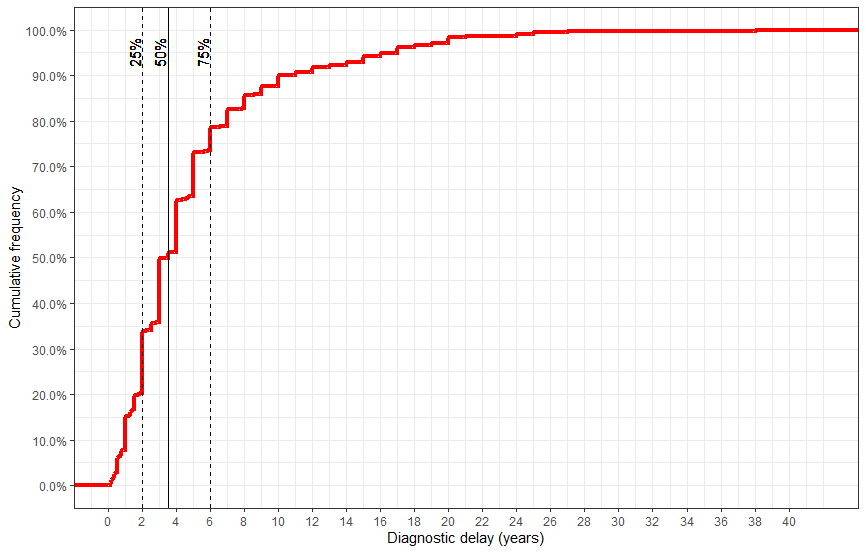

Supplement: Supplementary file 1 — Supplementary file1 (DOCX 129 kb) [file 223_2022_1005_MOESM1_ESM.docx]
