# Supplementary material for: Tumor-Induced Osteomalacia: A Systematic Clinical Review of 895 Cases
Source: Calcif Tissue Int. 2022 Jul 20;111(4):367–79. doi: 10.1007/s00223-022-01005-8 (PMC9474374; doi:10.1007/s00223-022-01005-8)
Supplement: Supplementary file 2 — Supplementary file2 (DOCX 66 kb) [file 223_2022_1005_MOESM2_ESM.docx]

**Tumor-induced osteomalacia: a systematic clinical review of 895 cases**

**Authors:** Ariadne Bosman^1^*, Andrea Palermo^2^*, Julien Vanderhulst^3^, Suzanne Jan De Beur^4^ , Seiji Fukumoto^5^, Salvatore Minisola^6^, Weibo Xia^7^, Jean-Jacques Body^3^, M. Carola Zillikens^1^.

*These authors contributed equally to this work

**Author Affiliations:**

^1^ Erasmus MC, University Medical Center Rotterdam, The Netherlands, Department of Internal Medicine.

^2^ Unit of Metabolic bone and thyroid disorders, Fondazione Policlinico Universitario Campus Bio-Medico, Rome, Italy

^3^ Department of Medicine, CHU Brugmann, Université Libre de Bruxelles (ULB), Brussels, Belgium.

^4^ Johns Hopkins University School of Medicine, Baltimore, MD, USA

^5^ Fujii Memorial Institute of Medical Sciences, Institute of Advanced Medical Sciences, Tokushima University, Tokushima, Japan

^6^ Department of Clinical, Internal, Anesthesiological and Cardiological Sciences, "Sapienza" Rome University, 00161 Rome, Italy

^7^ Department of Endocrinology, Key Laboratory of Endocrinology, The National Commission of Health, Peking Union Medical College Hospital, Chinese Academy of Medical Sciences, Beijing, China

**Corresponding author:**

M. Carola Zillikens

Erasmus MC, University Medical Center Rotterdam, The Netherlands, Department of Internal Medicine

Email: m.c.zillikens@erasmusmc.nl

**Online Resource 1**

List of articles included

1 McCance RA (1947) Osteomalacia with Looser's nodes (Milkman's syndrome) due to a raised resistance to vitamin D acquired about the age of 15 years. Q J Med 16:33-46

2 Prader A, Illig R, Uehlinger E, Stalder G (1959) [Rickets following bone tumor]. Helv Paediatr Acta 14:554-565

3 Yoshikawa S, Kawabata M, Hatsuyama Y, Hosokawa O, Fujita T (1964) Atypical Vitamin-D Resistant Osteomalacia. Report of a Case. J Bone Joint Surg Am 46:998-1007

4 (1965) Case records of the Massachusetts General Hospital. Case 38-1965. N Engl J Med 273:494-504

5 Salassa RM, Jowsey J, Arnaud CD (1970) Hypophosphatemic osteomalacia associated with "nonendocrine" tumors. N Engl J Med 283:65-70

6 Evans DJ, Azzopardi JG (1972) Distinctive tumours of bone and soft tissue causing acquired vitamin-D-resistant osteomalacia. Lancet 1:353-354

7 Olefsky J, Kempson R, Jones H, Reaven G (1972) "Tertiary" hyperparathyroidism and apparent "cure" of vitamin-D-resistant rickets after removal of an ossifying mesenchymal tumor of the pharynx. N Engl J Med 286:740-745

8 Pollack JA, Schiller AL, Crawford JD (1973) Rickets and myopathy cured by removal of a nonossifying fibroma of bone. Pediatrics 52:364-371

9 Moser CR, Fessel WJ (1974) Rheumatic manifestations of hypophosphatemia. Arch Intern Med 134:674-678

10 Willhoite DR (1975) ACQUIRED RICKETS AND SOLITARY BONE TUMOR: THE QUESTION OF A CAUSAL RELATIONSHIP. Clinical Orthopaedics and Related Research® 109:210-211

11 Linovitz RJ, Resnick D, Keissling P, Kondon JJ, Sehler B, Nejdl RJ, Rowe JH, Deftos LJ (1976) Tumor-induced osteomalacia and rickets: a surgically curable syndrome. Report of two cases. J Bone Joint Surg Am 58:419-423

12 Renton P, Shaw DG (1976) Hypophosphatemic osteomalacia secondary to vascular tumors of bone and soft tissue. Skeletal Radiology 1:21-24

13 Drezner MK, Feinglos MN (1977) Osteomalacia due to 1alpha,25-dihydroxycholecalciferol deficiency. Association with a giant cell tumor of bone. J Clin Invest 60:1046-1053

14 Wyman AL, Paradinas FJ, Daly JR (1977) Hypophosphataemic osteomalacia associated with a malignant tumour of the tibia: report of a case. J Clin Pathol 30:328-335

15 Yoshikawa S, Nakamura T, Takagi M, Imamura T, Okano K, Sasaki S (1977) Benign osteoblastoma as a cause of osteomalacia. A report of two cases. J Bone Joint Surg Br 59:279-286

16 Moncrieff MW, Brenton DP, Arthur LJH (1978) Case of tumour rickets. Archives of Disease in Childhood 53:740-745

17 Daniels RA, Weisenfeld I (1979) Tumorous phosphaturic osteomalacia. Report of a case associated with multiple hemangiomas of bone. Am J Med 67:155-159

18 Fukumoto Y, Tarui S, Tsukiyama K (1979) Tumor-induced vitamin D-resistant hypophosphatemic osteomalacia associated with proximal renal tubular dysfunction and 1,25-dihydroxyvitamin D deficiency. Journal of Clinical Endocrinology and Metabolism 49:873-878

19 Lejeune E, Bouvier M, Meunier P, Vauzelle JL, Deplante JP, David L, Llorca G, André-Fouet E (1979) [Osteomalacia of mesenchymatous tumors. Apropos of a new case] L'ostéomalacie des tumeurs mésenchymateuses. A propos d'une nouvelle observation. Rev Rhum Mal Osteoartic 46:187-193

20 Crouzet J, Camus JP, Gatti JM, Descamps H, Beraneck L (1980) [Hypophosphoremic osteomalacia and hemangiopericytoma of the vault of the cranium] Ostéomalacie hypophosphorémique et hémangiopéricytome de la voute du crâne. Rev Rhum Mal Osteoartic 47:523-528

21 Lyles KW, Berry WR, Haussler M (1980) Hypophosphatemia osteomalacia: Association with prostatic carcinoma. Annals of Internal Medicine 93:275-278

22 Sweet RA, Males JL, Hamstra AJ, DeLuca HF (1980) Vitamin D metabolite levels in oncogenic osteomalacia. Ann Intern Med 93:279-280

23 Asnes RS, Berdon WE, Bassett CA (1981) Hypophosphatemic rickets in an adolescent cured by excision of a nonossifying fibroma. Clin Pediatr (Phila) 20:646-648

24 Cramer SF, Aikawa M, Cebelin M (1981) Neurosecretory granules in small cell invasive carcinoma of the urinary bladder. Cancer 47:724-730

25 Nitzan DW, Marmary Y, Azaz B (1981) Mandibular tumor-induced muscular weakness and osteomalacia. Oral Surg Oral Med Oral Pathol 52:253-256

26 Parker MS, Klein I, Haussler MR, Mintz DH (1981) Tumor-induced osteomalacia. Evidence of a surgically correctable alteration in vitamin D metabolism. Jama 245:492-493

27 Camus JP, Crouzet J, Prier A, Koeger AC (1982) [Hypophosphoremic osteomalacia of connective tissue tumors] L'ostéomalacie hypophosphorémique des tumeurs du tissu conjonctif. Rev Rhum Mal Osteoartic 49:301-306

28 Nomura G, Koshino Y, Morimoto H, Kida H, Nomura S, Tamai K (1982) Vitamin D resistant hypophosphatemic osteomalacia associated with osteosarcoma of the mandible: report of a case. Jpn J Med 21:35-39

29 Agus ZS (1983) Oncogenic hypophosphatemic osteomalacia. Kidney Int 24:113-123

30 Linsey M, Smith W, Yamauchi H, Bernstein L (1983) Nasopharyngeal angiofibroma presenting as adult osteomalacia: case report and review of the literature. Laryngoscope 93:1328-1331

31 Martini A, Notarangelo LD, Barberis L (1983) Acquired vitamin D-resistant rickets caused by prolonged latency in appearance of bone tumor. American Journal of Diseases of Children 137:1205-1206

32 Taylor HC, Fallon MD, Velasco ME (1984) Oncogenic osteomalacia and inappropriate antidiuretic hormone secretion due to oat-cell carcinoma. Ann Intern Med 101:786-788

33 Jefferis AF, Taylor PC, Walsh-Waring GP (1985) Tumour-associated hypophosphataemic osteomalacia occurring in a patient with an odontogenic tumour of the maxilla. J Laryngol Otol 99:1011-1017

34 Leehey DJ, Ing TS, Daugirdas JT (1985) Fanconi syndrome associated with a non-ossifying fibroma of bone. Am J Med 78:708-710

35 Seshadri MS, Cornish CJ, Mason RS, Posen S (1985) Parathyroid hormone-like bioactivity in tumours from patients with oncogenic osteomalacia. Clin Endocrinol (Oxf) 23:689-697

36 Weidner N, Bar RS, Weiss D, Strottmann MP (1985) Neoplastic pathology of oncogenic osteomalacia/rickets. Cancer 55:1691-1705

37 Cotton GE, Van Puffelen P (1986) Hypophosphatemic osteomalacia secondary to neoplasia. J Bone Joint Surg Am 68:129-133

38 Furuya K, Isobe Y, Morita M (1986) [Hypertrophic osteoarthropathy and hypophosphatemic osteomalacia associated with tumor]. Gan To Kagaku Ryoho 13:2056-2064

39 Rico H, Fernandez-Miranda E, Sanz J (1986) Case report: Oncogenous osteomalacia: A new case secondary to a malignant tumor. Bone 7:325-329

40 Ryan WG, Gitelis S, Charters JR (1986) Studies in a patient with tumor-induced hypophosphatemic osteomalacia. Calcif Tissue Int 38:358-362

41 McClure J, Smith PS (1987) Oncogenic osteomalacia. J Clin Pathol 40:446-453

42 Prowse M, Brooks PM (1987) Oncogenic hypophosphatemic osteomalacia associated with a giant cell tumour of a tendon sheath. Aust N Z J Med 17:330-332

43 Reid IR, Teitelbaum SL, Dusso A, Whyte MP (1987) Hypercalcemic hyperparathyroidism complicating oncogenic osteomalacia. Effect of successful tumor resection on mineral homeostasis. Am J Med 83:350-354

44 Siris ES, Clemens TL, Dempster DW, Shane E, Segre GV, Lindsay R, Bilezikian JP (1987) Tumor-induced osteomalacia. Kinetics of calcium, phosphorus, and vitamin D metabolism and characteristics of bone histomorphometry. Am J Med 82:307-312

45 Sparagana M (1987) Tumor-induced osteomalacia: long-term follow-up of two patients cured by removal of their tumors. J Surg Oncol 36:198-205

46 Miyauchi A, Fukase M, Tsutsumi M, Fujita T (1988) Hemangiopericytoma-induced osteomalacia: tumor transplantation in nude mice causes hypophosphatemia and tumor extracts inhibit renal 25-hydroxyvitamin D 1-hydroxylase activity. J Clin Endocrinol Metab 67:46-53

47 Taylor HC, Santa-Cruz D, Teitelbaum SL, Bergfeld MA, Whyte MP (1988) Assessment of calcitriol and inorganic phosphate therapy before cure of oncogenous osteomalacia by resection of a mixed mesenchymal tumor. Bone 9:37-43

48 McGuire MH, Merenda JT, Etzkorn JR, Sundaram M (1989) Oncogenic osteomalacia. A case report. Clin Orthop Relat Res:305-308

49 Nitzan DW, Horowitz AT, Darmon D, Friedlaender MM, Rubinger D, Stein P, Bab I, Popovtzer MM, Silver J (1989) Oncogenous osteomalacia: a case study. Bone Miner 6:191-197

50 Nuovo MA, Dorfman HD, Sun CC, Chalew SA (1989) Tumor-induced osteomalacia and rickets. Am J Surg Pathol 13:588-599

51 Segre GV, Dickersin GR, Ehana S, Scully RE, Weinstein DF, Jacobs EE, Kronenberg HM, Pier AS (1989) A 63-YEAR-OLD MAN WITH OSTEOMALACIA AND THE LATER DEVELOPMENT OF A RIGHT NASAL MASS - BENIGN MIXED MESENCHYMAL NEOPLASM WITH HEMANGIOPERICYTOMA COMPONENT INVOLVING NASAL CAVITY AND ETHMOID SINUS, WITH ONCOGENIC OSTEOMALACIA. New England Journal of Medicine 321:1812-1821

52 Uchida H, Yokoyama S, Kashima K, Nakayama I, Shimizu K, Masumi S (1991) Oncogenic vitamin D resistant hypophosphatemic osteomalacia (benign ossifying mesenchymal tumor of bone): case report. Jpn J Clin Oncol 21:218-226

53 Stone MD, Quincey C, Hosking DJ (1992) A neuroendocrine cause of oncogenic osteomalacia. J Pathol 167:181-185

54 Amir G, Boneh A, Tochner Z, Bar Ziv J (1993) Widespread hemangiomatosis of bone associated with rickets: recovery after irradiation. J Pediatr 123:269-272

55 McMurtry CT, Godschalk M, Malluche HH, Geng Z, Adler RA (1993) Oncogenic osteomalacia associated with metastatic prostate carcinoma: case report and review of the literature. J Am Geriatr Soc 41:983-985

56 Cai Q, Hodgson SF, Kao PC, Lennon VA, Klee GG, Zinsmiester AR, Kumar R (1994) Brief report: inhibition of renal phosphate transport by a tumor product in a patient with oncogenic osteomalacia. N Engl J Med 330:1645-1649

57 Cehreli C, Alakavuklar MN, Cavdar C, Basdemir G, Undar B, Akkoc N, Payzin B, Oztop F (1994) Oncogenous osteomalacia--report of a case. Acta Oncol 33:975-976

58 Robin N, Gill G, Vanheyningen C, Fraser W (1994) A SMALL-CELL BRONCHOGENIC-CARCINOMA ASSOCIATED WITH TUMORAL HYPOPHOSPHATEMIA AND INAPPROPRIATE ANTIDIURESIS. Postgraduate Medical Journal 70:746-748

59 Eyskens B, Proesmans W, Van Damme B, Lateur L, Bouillon R, Hoogmartens M (1995) Tumour-induced rickets: a case report and review of the literature. Eur J Pediatr 154:462-468

60 Kim SH OB, Hang EH, Lee SR (1995) [Tumor-induced hypophosphatemic osteomalacia: report of three cases]. Korean Acad Oral Maxillofac Radiol:141-151

61 Lee HK, Sung WW, Solodnik P, Shimshi M (1995) Bone scan in tumor-induced osteomalacia. J Nucl Med 36:247-249

62 Schapira D, Ben Izhak O, Nachtigal A, Burstein A, Shalom RB, Shagrawi I, Best LA (1995) Tumor-induced osteomalacia. Semin Arthritis Rheum 25:35-46

63 Wilkins GE, Granleese S, Hegele RG, Holden J, Anderson DW, Bondy GP (1995) Oncogenic osteomalacia: evidence for a humoral phosphaturic factor. J Clin Endocrinol Metab 80:1628-1634

64 Chalew SA, Lovchik JC, Brown CM, Sun CC (1996) Hypophosphatemia induced in mice by transplantation of a tumor-derived cell line from a patient with oncogenic rickets. J Pediatr Endocrinol Metab 9:593-597

65 Kim YG, Choi YS, Lee SC, Ryu DM (1996) Tumor-induced osteomalacia associated with lesions in the oral and maxillofacial region: report of two cases. J Oral Maxillofac Surg 54:1352-1357

66 Nelson AE, Namkung HJ, Patava J, Wilkinson MR, Chang AC, Reddel RR, Robinson BG, Mason RS (1996) Characteristics of tumor cell bioactivity in oncogenic osteomalacia. Mol Cell Endocrinol 124:17-23

67 Tsujimura T, Sakaguchi K, Aozasa K (1996) Phosphaturic mesenchymal tumor, mixed connective tissue variant (oncogenic osteomalacia). Pathol Int 46:238-241

68 Francois S, Lefort G, PoliMerol ML, Gaillard D, Roussel B, Sulmont V, Daoud S (1997) Bone tumor exeresis for secondary rickets treatment: A case report. Revue De Chirurgie Orthopedique Et Reparatrice De L Appareil Moteur 83:387-392

69 Hirano T, Tanizawa T, Endo N, Takahashi HE, Morita T (1997) Oncogenic osteomalacia: Pre- and postoperative histomorphometric studies. Journal of Bone and Mineral Metabolism 15:227-231

70 Shane E, Parisien M, Henderson JE, Dempster DW, Feldman F, Hardy MA, Tohme JF, Karaplis AC, Clemens TL (1997) Tumor-induced osteomalacia: clinical and basic studies. J Bone Miner Res 12:1502-1511

71 Yang IM, Park YK, Hyun YJ, Kim DY, Woo JT, Kim SW, Kim JW, Kim YS, Choi YK (1997) Oncogenic osteomalacia caused by a phosphaturic mesenchymal tumor of the oral cavity: a case report. Korean J Intern Med 12:89-95

72 Kats S, Markusse HM, Vecht CJ (1998) Severe muscle weakness secondary to paraneoplastic hypophosphatemia in neuroblastoma. Netherlands Journal of Medicine 53:207-211

73 Nelson AE, Mason RS, Hogan JJ, Diamond T, Robinson BG (1998) Tumor expression studies indicate that HEM-1 is unlikely to be the active factor in oncogenic osteomalacia. Bone 23:549-553

74 Ohta H, Nakaishi S, Oki S, Simizu M, Watanabe H, Fujikawa S, Nakade M, Watanabe H, Kohno K, Shintaku M (1998) Bone scintigraphy in a case of oncogenous osteomalacia. Clin Nucl Med 23:467-469

75 Fukumoto S, Takeuchi Y, Nagano A, Fujita T (1999) Diagnostic utility of magnetic resonance imaging skeletal survey in a patient with oncogenic osteomalacia. Bone 25:375-377

76 Gascón A, Cobeta-Garcia JC, Iglesias E, Lázaro JM, Muniesa JA (1999) Oncogenic osteomalacia in a patient with a fibrocystic nodule of the breast. Nephrol Dial Transplant 14:1561-1563

77 Hasegawa T, Shimoda T, Yokoyama R, Beppu Y, Hirohashi S, Maeda S (1999) Intracortical osteoblastic osteosarcoma with oncogenic rickets. Skeletal Radiol 28:41-45

78 Lim SK SY, Lee HC,Huh KB,Park KH,Kim KR,Hong SW,Nam JH,Yook JI,Choi BJ,Kim MK,Shin KH (1999) A Case of Osteosarcoma induced Oncogenic Osteomalacia Detected by MRI. J Korean Soc Endocrinol:401-409

79 Ohashi K, Ohnishi T, Ishikawa T, Tani H, Uesugi K, Takagi M (1999) Oncogenic osteomalacia presenting as bilateral stress fractures of the tibia. Skeletal Radiol 28:46-48

80 Zura RD, Minasi JS, Kahler DM (1999) Tumor-induced osteomalacia and symptomatic looser zones secondary to mesenchymal chondrosarcoma. J Surg Oncol 71:58-62

81 Clunie GP, Fox PE, Stamp TC (2000) Four cases of acquired hypophosphataemic ('oncogenic') osteomalacia. Problems of diagnosis, treatment and long-term management. Rheumatology (Oxford) 39:1415-1421

82 Mátyus J, Szebenyi B, Rédl P, Mikita J, Gáspár L, Haris A, Radó J, Kakuk G (2000) [Hypophosphatemic oncogenic osteomalacia] Hypophosphataemiával járó onkogén osteomalacia. Orv Hetil 141:2785-2788

83 Reyes-Múgica M, Arnsmeier SL, Backeljauw PF, Persing J, Ellis B, Carpenter TO (2000) Phosphaturic mesenchymal tumor-induced rickets. Pediatr Dev Pathol 3:61-69

84 Sandhu FA, Martuza RL (2000) Craniofacial hemangiopericytoma associated with oncogenic osteomalacia: case report. J Neurooncol 46:241-247

85 John MR, Wickert H, Zaar K, Jonsson KB, Grauer A, Ruppersberger P, Schmidt-Gayk H, Murer H, Ziegler R, Blind E (2001) A case of neuroendocrine oncogenic osteomalacia associated with a PHEX and fibroblast growth factor-23 expressing sinusidal malignant schwannoma. Bone 29:393-402

86 Kawai Y, Morimoto S, Sakaguchi K, Yoshino H, Yotsui T, Hirota S, Inohara H, Nakagawa T, Hattori K, Kubo T, Yang J, Fujiwara N, Ogihara T (2001) Oncogenic osteomalacia secondary to nasal tumor with decreased urinary excrretion of cAMP. Journal of Bone and Mineral Metabolism 19:61-64

87 Nelson AE, Mason RS, Robinson BG, Hogan JJ, Martin EA, Ahlström H, Aström G, Larsson T, Jonsson K, Wibell L, Ljunggren O (2001) Diagnosis of a patient with oncogenic osteomalacia using a phosphate uptake bioassay of serum and magnetic resonance imaging. Eur J Endocrinol 145:469-476

88 Park JM, Woo YK, Kang MI, Kang CS, Hahn ST (2001) Oncogenic osteomalacia associated with soft tissue chondromyxoid fibroma. Eur J Radiol 39:69-72

89 Rhee Y, Lee JD, Shin KH, Lee HC, Huh KB, Lim SK (2001) Oncogenic osteomalacia associated with mesenchymal tumour detected by indium-111 octreotide scintigraphy. Clin Endocrinol (Oxf) 54:551-554

90 Sakamoto A, Oda Y, Nagayoshi Y, Iwakiri K, Tamiya S, Iwamoto Y, Tsuneyoshi M (2001) Glomangiopericytoma causing oncogenic osteomalacia. A case report with immunohistochemical analysis. Arch Orthop Trauma Surg 121:104-108

91 Sato K, Obara T, Yamazaki K, Kanbe M, Nakajima K, Yamada A, Yanagisawa T, Kato Y, Nishikawa T, Takano K (2001) Somatic mutations of the MEN1 gene and microsatellite instability in a case of tertiary hyperparathyroidism occurring during high phosphate therapy for acquired, hypophosphatemic osteomalacia. J Clin Endocrinol Metab 86:5564-5571

92 Seufert J, Ebert K, Müller J, Eulert J, Hendrich C, Werner E, Schuüze N, Schulz G, Kenn W, Richtmann H, Palitzsch KD, Jakob F (2001) Octreotide therapy for tumor-induced osteomalacia. N Engl J Med 345:1883-1888

93 Terek RM, Royster AP, Nielsen GP, Ebb D, Mankin K (2001) A 14-year-old boy with abnormal bones and a sacral mass - Osteosarcoma of the sacrum. Oncogenic rickets. New England Journal of Medicine 345:903-908

94 Furco A, Roger M, Mouchet B, Richard O, Martinache X, Fur A (2002) Osteomalacia cured by surgery. Eur J Intern Med 13:67-69

95 Garcia CA, Spencer RP (2002) Bone and In-111 octreotide imaging in oncogenic osteomalacia: a case report. Clin Nucl Med 27:582-583

96 Jan De Beur SM, Finnegan RB, Vassiliadis J, Cook B, Barberio D, Estes S, Manavalan P, Petroziello J, Madden SL, Cho JY, Kumar R, Levine MA, Schiavi SC (2002) Tumors associated with oncogenic osteomalacia express genes important in bone and mineral metabolism. Journal of Bone and Mineral Research 17:1102-1110

97 Jan de Beur SM, Streeten EA, Civelek AC, McCarthy EF, Uribe L, Marx SJ, Onobrakpeya O, Raisz LG, Watts NB, Sharon M, Levine MA (2002) Localisation of mesenchymal tumours by somatostatin receptor imaging. Lancet 359:761-763

98 Lui CY, Khoo R, Law TC, Chong SF (2002) Case report. Tumour-induced osteomalacia in a patient with osseous haemangioma. Clin Radiol 57:1125-1127

99 Moran M, Paul A (2002) Octreotide scanning in the detection of a mesenchymal tumour in the pubic symphysis causing hypophosphataemic osteomalacia. Int Orthop 26:61-62

100 Reis-Filho JS, Paiva ME, Lopes JM (2002) Pathologic quiz case. A 36-year-old woman with muscle pain and weakness. Phosphaturic mesenchymal tumor (mixed connective tissue variant)/oncogenic osteomalacia. Arch Pathol Lab Med 126:1245-1246

101 Yamazaki Y, Okazaki R, Shibata M, Hasegawa Y, Satoh K, Tajima T, Takeuchi Y, Fujita T, Nakahara K, Yamashita T, Fukumoto S (2002) Increased circulatory level of biologically active full-length FGF-23 in patients with hypophosphatemic rickets/osteomalacia. J Clin Endocrinol Metab 87:4957-4960

102 Chun KA, Cho IH, Won KJ, Lee HW, Choi JH, Ahn JC, Shin DS (2003) Osteoblastoma as a cause of osteomalacia assessed by bone scan. Ann Nucl Med 17:411-414

103 Dissanayake AM, Wilson JL, Holdaway IM, Reid IR (2003) Oncogenic osteomalacia: culprit tumour detection whole body magnetic resonance imaging. Intern Med J 33:615-616

104 Fuentealba C, Pinto D, Ballesteros F, Pacheco D, Boettiger O, Soto N, Fernandez W, Gabler F, Gonzales G, Reginato AJ (2003) Oncogenic hypophosphatemic osteomalacia associated with a nasal hemangiopericytoma. J Clin Rheumatol 9:373-379

105 Heller HJ, Zerwekh JE, Odvina CV, Raisanen J (2003) Physiologic evaluation of a patient with oncogenic osteomalacia before and after cure. Journal of Bone and Mineral Research 18:S416-S416

106 Hoogendoorn EH, White KE, Econs MJ, Hermus AR (2003) Hypophosphatemia, osteomalacia and proximal muscle weakness treated by surgery. Clin Endocrinol (Oxf) 58:796-797

107 Nelson AE, Bligh RC, Mirams M, Gill A, Au A, Clarkson A, Jüppner H, Ruff S, Stalley P, Scolyer RA, Robinson BG, Mason RS, Bligh PC (2003) Clinical case seminar: Fibroblast growth factor 23: a new clinical marker for oncogenic osteomalacia. J Clin Endocrinol Metab 88:4088-4094

108 Sapunar J, Roa JC, Moscoso S (2003) [Reversion of hypophosphatemia after the excision of a composite hemangioendothelioma in the great toe] Hipofosfatemia revertida al extirpar hemangioendotelioma compuesto del dedo mayor del pie. Rev Med Chil 131:909-914

109 Kimizuka T, Ozaki Y, Sumi Y (2004) Usefulness of 201Tl and 99mTc MIBI scintigraphy in a case of oncogenic osteomalacia. Ann Nucl Med 18:63-67

110 Reis-Filho JS, Paiva ME, Lopes JM (2004) August 2003: 47-year-old female with a 7-year history of osteomalacia and hypophosphatemia. Brain Pathol 14:111-112, 115

111 Shulman DI, Hahn G, Benator R, Washington K, White KE, Farber J, Econs MJ (2004) Tumor-induced rickets: usefulness of MR gradient echo recall imaging for tumor localization. J Pediatr 144:381-385

112 Takeuchi Y, Suzuki H, Ogura S, Imai R, Yamazaki Y, Yamashita T, Miyamoto Y, Okazaki H, Nakamura K, Nakahara K, Fukumoto S, Fujita T (2004) Venous sampling for fibroblast growth factor-23 confirms preoperative diagnosis of tumor-induced osteomalacia. J Clin Endocrinol Metab 89:3979-3982

113 Ungari C, Rocchi G, Rinna C, Agrillo A, Lattanzi A, Pagnoni M (2004) Hypophosphaturic mesenchymal tumor of the ethmoid associated with oncogenic osteomalacia. J Craniofac Surg 15:523-527

114 Ward LM, Rauch F, White KE, Filler G, Matzinger MA, Letts M, Travers R, Econs MJ, Glorieux FH (2004) Resolution of severe, adolescent-onset hypophosphatemic rickets following resection of an FGF-23-producing tumour of the distal ulna. Bone 34:905-911

115 Carpenter TO, Ellis BK, Insogna KL, Philbrick WM, Sterpka J, Shimkets R (2005) Fibroblast growth factor 7: an inhibitor of phosphate transport derived from oncogenic osteomalacia-causing tumors. J Clin Endocrinol Metab 90:1012-1020

116 Colt E, Gopan T, Chong HS (2005) Oncogenic osteomalacia cured by removal of an organized hematoma. Endocr Pract 11:190-193

117 Dupond JL, Mahammedi H, Prié D, Collin F, Gil H, Blagosklonov O, Ricbourg B, Meaux-Ruault N, Kantelip B (2005) Oncogenic osteomalacia: diagnostic importance of fibroblast growth factor 23 and F-18 fluorodeoxyglucose PET/CT scan for the diagnosis and follow-up in one case. Bone 36:375-378

118 Ludwig J, Mechtersheimer G, Haberkorn U, Nawroth P, Schilling T, Kasperk C (2005) Oncogenic osteomalacia in a 49-year old female. Deutsche Medizinische Wochenschrift 130:206-209

119 Menon VU, Nair V, Kumar H, Rajanikanth U (2005) Oncogenic osteomalacia. Endocrinologist 15:65-68

120 Smith RM, Ostor AJK, Teir J, Adlam DM, Crisp AJ (2005) 308. ONCOGENIC HYPOPHOSPHATAEMIC OSTEOMALACIA SECONDARY TO A HUMERAL OSTEOCHONDROMA PRESENTING WITH ACUTE PYROPHOSPHATE ARTHROPATHY OF THE TEMPOROMANDIBULAR JOINT. Rheumatology 44

121 Zimering MB, Caldarella FA, White KE, Econs MJ (2005) Persistent tumor-induced osteomalacia confirmed by elevated postoperative levels of serum fibroblast growth factor-23 and 5-year follow-up of bone density changes. Endocr Pract 11:108-114

122 Cheung FM, Ma L, Wu WC, Siu TH, Choi PT, Tai YP (2006) Oncogenic osteomalacia associated with an occult phosphaturic mesenchymal tumour: clinico-radiologico-pathological correlation and ultrastructural studies. Hong Kong Med J 12:319-321

123 Imel EA, Peacock M, Pitukcheewanont P, Heller HJ, Ward LM, Shulman D, Kassem M, Rackoff P, Zimering M, Dalkin A, Drobny E, Colussi G, Shaker JL, Hoogendoorn EH, Hui SL, Econs MJ (2006) Sensitivity of fibroblast growth factor 23 measurements in tumor-induced osteomalacia. Journal of Clinical Endocrinology and Metabolism 91:2055-2061

124 Inokuchi G, Tanimoto H, Ishida H, Sugimoto T, Yamauchi M, Miyauchi A, Nibu K (2006) A paranasal tumor associated with tumor-induced osteomalacia. Laryngoscope 116:1930-1933

125 Kaylie DM, Jackson CG, Gardner EK (2006) Oncogenic osteomalacia caused by phosphaturic mesenchymal tumor of the temporal bone. Otolaryngol Head Neck Surg 135:653-654

126 Koriyama N, Nishimoto K, Kodama T, Nakazaki M, Kurono Y, Yoshida H, Tei C (2006) Oncogenic osteomalacia in a case with a maxillary sinus mesenchymal tumor. Am J Med Sci 332:142-147

127 Ladha SS, Whitaker MD, Bosch EP (2006) Oncogenic osteomalacia: muscular weakness and multiple fractures. Neurology 67:364-365

128 Sahnoune I, Tazi-Mezalek Z, Essaadouni L, Harmouche H, Adnaoui M, Aouni M, Maaouni A, Ismael F, Kettani F (2006) Oncogenic osteomalacia in a patient with hemangioma: A clinical diagnosis [2]. Joint Bone Spine 73:115-118

129 Shelekhova KV, Kazakov DV, Hes O, Treska V, Michal M (2006) Phosphaturic mesenchymal tumor (mixed connective tissue variant): a case report with spectral analysis. Virchows Arch 448:232-235

130 Vandergheynst F, Van Dorpe J, Goldman S, Decaux G (2006) Increased 18F fluorodeoxyglucose uptake of a vertebral hemangioma responsible for oncogenic osteomalacia. Eur J Intern Med 17:223

131 Yoshioka K, Nagata R, Ueda M, Yamaguchi T, Konishi Y, Hosoi M, Inoue T, Yamanaka K, Iwai Y, Sato T (2006) Phosphaturic mesenchymal tumor with symptoms related to osteomalacia that appeared one year after tumorectomy. Intern Med 45:1157-1160

132 Ahn JM, Kim HJ, Cha CM, Kim J, Yim SG, Kim HJ (2007) Oncogenic osteomalacia: induced by tumor, cured by surgery. Oral Surg Oral Med Oral Pathol Oral Radiol Endod 103:636-641

133 Bilen H, Akcay G, Polat P, Akcay M, Yildirim O (2007) Tumor induced osteomalacia caused by haemangioma of the acetabular surface. In: 9th European Congress of Endocrinology. BioScientifica

134 Cotant CL, Rao PS (2007) Elevated fibroblast growth factor 23 in a patient with metastatic prostate cancer and hypophosphatemia. Am J Kidney Dis 50:1033-1036

135 Dewitt CA, Collins MT, Cowen EW (2007) Diffuse pain, hypophosphatemia, and a subcutaneous nodule. J Am Acad Dermatol 57:509-512

136 Elston MS, Stewart IJ, Clifton-Bligh R, Conaglen JV (2007) A case of oncogenic osteomalacia with preoperative secondary hyperparathyroidism: description of the biochemical response of FGF23 to octreotide therapy and surgery. Bone 40:236-241

137 Filipponi P, Cristallini S, Policani G, Nicasi A, Bracaccia M (2007) Oncogenic osteomalacia caused by a FGF 23-producing hemangiopericytoma detected with octreotide scan. Calcified Tissue International 80:S36-S36

138 Gershinsky M, Croitoru S, Dickstein G, Bardicef O, Gelman R, Barmeir E (2007) Imaging of oncogenic osteomalacia. Isr Med Assoc J 9:566-567

139 Halperin F, Anderson RJ, Mulder JE (2007) Tumor-induced osteomalacia: the importance of measuring serum phosphorus levels. Nat Clin Pract Endocrinol Metab 3:721-725

140 Harbeck B, Schoecklmann H, Seekamp A, Czech N, Moenig H (2007) Tumour-induced hypophosphataemic osteomalacia. In: Calcified tissue international. SPRINGER 233 SPRING STREET, NEW YORK, NY 10013 USA, p S64-S64

141 Hesse E, Moessinger E, Rosenthal H, Laenger F, Brabant G, Petrich T, Gratz KF, Bastian L (2007) Oncogenic osteomalacia: exact tumor localization by co-registration of positron emission and computed tomography. J Bone Miner Res 22:158-162

142 Hesse E, Rosenthal H, Bastian L (2007) Radiofrequency ablation of a tumor causing oncogenic osteomalacia [17]. New England Journal of Medicine 357:422-424

143 Jacob JJ, Finny P, Thomas M, Thomas N, John M (2007) Oncogenic osteomalacia. J Assoc Physicians India 55:231-233

144 Kaul M, Silverberg M, Dicarlo EF, Schneider R, Bass AR, Erkan D (2007) Tumor-induced osteomalacia. Clin Rheumatol 26:1575-1579

145 Khosravi A, Cutler CM, Kelly MH, Chang R, Royal RE, Sherry RM, Wodajo FM, Fedarko NS, Collins MT (2007) Determination of the elimination half-life of fibroblast growth factor-23. J Clin Endocrinol Metab 92:2374-2377

146 Oka M, Kamo T, Sasaki E, Kaji H, Nishizawa H, Imanishi Y, Nishigori C (2007) A case of phosphaturic mesenchymal tumour (mixed connective tissue variant) that developed in the subcutaneous tissue of a patient with oncogenic osteomalacia and produced fibroblast growth factor 23 [9]. British Journal of Dermatology 157:198-200

147 Paul S, Kurtz M, Mentzer SJ (2007) Osteomalacia associated with a fibroblast growth factor-23 secreting chest wall tumor. J Thorac Cardiovasc Surg 134:803-805

148 Tournis S, Samdanis V, Economopoulos D, Giannikou P, Lyritis GP (2007) Autonomous hyperparathyroidism following long-term phosphate treatment for tumor-induced osteomalacia: Case report and review of the literature. Endocrinologist 17:263-266

149 (2008) Erratum: Oncogenic osteomalacia, a rare paraneoplastic syndrome due to phosphate wasting - A case report and review of the literature (Clinical Nephrology (2008) vol. 70 (5) (431-438)). Clinical Nephrology 70:564

150 Angelou A, Afrantou T, Paschalidou M, Milonas I, Tascos N (2008) Gait impairment and hypophosphataemia. A case of acquired hypophosphatemic "oncogenic" osteomalacia in a 40-year-old patient. Journal of Neurology 255:172-172

151 Bahrami A, Haaland DA, Churchill DN, Pritzker KPH, Adachi JD (2008) Oncogenic osteomalacia. A case report and literature review. Journal of Rheumatology 35:1200-1201

152 Biagini GLK, Coutinho PR, Jonasson TH, Ueda CE, Gama RR (2008) Oncogenic osteomalacia: Localization of underlying peripheral tumor with 99mTc-sestamibi scintigraphy. Arquivos Brasileiros de Endocrinologia e Metabologia 52:1505-1509

153 Bullmann C, Benker G, Rosien U, Delling G, Siggelkow H, Schulte HM (2008) [Hypophosphatemic osteomalacia] Hypophosphatämische Osteomalazie. Med Klin (Munich) 103:671-675

154 Chua SC, O'Connor SR, Wong WL, Ganatra RH (2008) Case report: Solitary plasmacytoma of bone with oncogenic osteomalacia: recurrence of tumour confirmed by PET/CT. A case report with a review of the radiological literature. Br J Radiol 81:e110-114

155 Duet M, Kerkeni S, Sfar R, Bazille C, Lioté F, Orcel P (2008) Clinical impact of somatostatin receptor scintigraphy in the management of tumor-induced osteomalacia. Clin Nucl Med 33:752-756

156 Harish S, Jurriaans E, Jan E, Sur M, Colterjohn N (2008) Giant cell tumour of soft tissue causing oncogenic osteomalacia: report demonstrating the use of octreotide scintigraphy in tumour localization. Clin Radiol 63:101-107

157 Hoshino C, Satoh N, Sugawara S, Kuriyama C, Kikuchi A, Ohta M (2008) Sporadic adult-onset hypophosphatemic osteomalacia caused by excessive action of fibroblast growth factor 23. Internal Medicine 47:453-457

158 Kenealy H, Holdaway I, Grey A (2008) Occult nasal sinus tumours causing oncogenic osteomalacia. Eur J Intern Med 19:516-519

159 Lewiecki EM, Urig EJ, Jr., Williams RC, Jr. (2008) Tumor-induced osteomalacia: lessons learned. Arthritis Rheum 58:773-777

160 Nasu T, Kurisu S, Matsuno S, Tatsumi K, Kakimoto T, Kobayashi M, Nakano Y, Wakasaki H, Furuta H, Nishi M, Sasaki H, Suzuki H, Ito N, Fukumoto S, Nanjo K (2008) Tumor-induced hypophosphatemic osteomalacia diagnosed by the combinatory procedures of magnetic resonance imaging and venous sampling for FGF23. Internal Medicine 47:957-961

161 Ogura E, Kageyama K, Fukumoto S, Yagihashi N, Fukuda Y, Kikuchi T, Masuda M, Suda T (2008) Development of tumor-induced osteomalacia in a subcutaneous tumor, defined by venous blood sampling of fibroblast growth factor-23. Internal Medicine 47:637-641

162 Poiana C, Carsote M, Chirita C, Hortopan D, Ioachim D, Goldstein A (2008) Medullar Carcinoma of the Thyroid and Anomalies of the Calcium Metabolism: Case Report. Journal of Bone and Mineral Research 23:S302-S302

163 Policarpio-Nicolas ML, Abbott TE, Dalkin AC, Bennett-Wick J, Frierson HF, Jr. (2008) Phosphaturic mesenchymal tumor diagnosed by fine-needle aspiration and core biopsy: a case report and review of literature. Diagn Cytopathol 36:115-119

164 Ratanasuwan T, Chetsurakarn S, Ongphiphadhanakul B, Damrongkitchaiporn S (2008) A case report of tumor-induced osteomalacia: Eight year followed-up. Journal of the Medical Association of Thailand 91:1900-1903

165 van Boekel G, Ruinemans-Koerts J, Joosten F, Dijkhuizen P, van Sorge A, de Boer H (2008) Tumor producing fibroblast growth factor 23 localized by two-staged venous sampling. Eur J Endocrinol 158:431-437

166 Westerberg PA, Olauson H, Toss G, Wikström B, Morales O, Linde T, Jonsson K, Ljunggren O, Larsson TE (2008) Preoperative tumor localization by means of venous sampling for fibroblast growth factor-23 in a patient with tumor-induced osteomalacia. Endocr Pract 14:362-367

167 Woznowski M, Quack I, Stegbauer J, Büchner N, Rump LC, Schieren G (2008) Oncogenic osteomalacia, a rare paraneoplastic syndrome due to phosphate wasting--a case report and review of the literature. Clin Nephrol 70:431-438

168 Brunschweiler B, Guedj N, Lenoir T, Faillot T, Rillardon L, Guigui P (2009) Oncogenous osteomalacia and myopericytoma of the thoracic spine: a case report. Spine (Phila Pa 1976) 34:E857-860

169 Findeisen HM, Auernhammer CJ, Parhofer KG, Herrmann KA, la Fougere C, Weiler C, Bartl R, Koch E (2009) [Reduced bone density and bone pain :osteomalacia with hypophospatemia and hypophosphaturia] Erniedrigte Knochendichte und Knochenschmerzen : Osteomalazie mit Hypophosphatämie und Hyperphosphaturie. Internist (Berl) 50:1402-1407

170 Ghosh S, Sinha R, Bandyopadhyay R, Malhotra M (2009) Oncogenous osteomalacia. J Cancer Res Ther 5:210-212

171 Gore MO, Welch BJ, Geng W, Kabbani W, Maalouf NM, Zerwekh JE, Moe OW, Sakhaee K (2009) Renal phosphate wasting due to tumor-induced osteomalacia: a frequently delayed diagnosis. Kidney Int 76:342-347

172 Gupta R, Sharma A, Ksh A, Khadgawat R, Dinda AK (2009) PHOSPHATURIC MESENCHYMAL TUMOR OF THE SINONASAL TRACT. Acta Endocrinologica-Bucharest 5:537-542

173 Harbeck B, Schöcklmann H, Seekamp A, Czech N, Mönig H (2009) Tumor-induced osteomalacia: successful treatment by radio-guided tumor surgery. J Clin Rheumatol 15:31-34

174 Iba K, Sasaki K, Takada J, Minowa T, Ito N, Fukumoto S, Hasegawa T, Wada T, Yamashita T (2009) Improvement of severe bone pain and activities of daily living in a patient with tumor-induced osteomalacia following the resection of a FGF23-producing- tumor in the oral cavity. Journal of Orthopaedics 6(4)e11

175 Khadgawat R, Singh Y, Kansara S, Tandon N, Bal C, Seith A, Kotwal P (2009) PET/CT localisation of a scapular haemangiopericytoma with tumour-induced osteomalacia. Singapore Med J 50:e55-57

176 Müssig K, Öksüz MÖ, Pfannenberg C, Adam P, Zustin J, Beckert S, Petersenn S (2009) Somatostatin receptor expression in an epitheloid hemangioma causing oncogenic osteomalacia. Journal of Clinical Endocrinology and Metabolism 94:4123-4124

177 Nair LT, Dodd L, Weber TJ (2009) Following the Forgotten Phosphorus. American Journal of Medicine 122:1093-1095

178 Nawrot-Wawrzyniak K, Varga F, Nader A, Roschger P, Sieghart S, Zwettler E, Roetzer KM, Lang S, Weinkamer R, Klaushofer K, Fratzl-Zelman N (2009) Effects of tumor-induced osteomalacia on the bone mineralization process. Calcif Tissue Int 84:313-323

179 Pirola E, Vergani F, Casiraghi P, Leone EB, Guerra P, Sganzerla EP (2009) Oncogenic osteomalacia caused by a phosphaturic mesenchymal tumor of the thoracic spine: Case report. Journal of Neurosurgery: Spine 10:329-333

180 Radaideh ARM, Jaradat D, Abu-Kalaf MM, Nusier MK (2009) Resolution of severe oncogenic hypophosphatemic osteomalacia after resection of a deeply located soft-tissue tumour. Current Oncology 16:87-90

181 Reilly BM, Hart PD, Mascarell S, Chatrath H (2009) A Question Well Put. New England Journal of Medicine 360:1446-1451

182 Romualdo-Silva DD, Silva BCC, Caetano CV, Tibúrcio AMFP, Nunes MB, Chagas SAP, Polito ETL, Ferreira AR, Purisch S (2009) Tumor-induced osteomalacia: A case report. Arquivos Brasileiros de Endocrinologia e Metabologia 53:378-382

183 Savage CR, Zimmer LA (2009) Oncogenic osteomalacia from pterygopalatine fossa mass. J Laryngol Otol 123:1052-1054

184 Sciubba DM, Petteys RJ, Shakur SF, Gokaslan ZL, McCarthy EF, Collins MT, McGirt MJ, Hsieh PC, Nelson CS, Wolinsky JP (2009) En bloc spondylectomy for treatment of tumor-induced osteomalacia: Case report. Journal of Neurosurgery: Spine 11:600-604

185 Shires R, Magan A, Huddle KRL (2009) Tumour-induced osteomalacia: A curable condition. Journal of Endocrinology, Metabolism and Diabetes of South Africa 14:143-144

186 Uramoto N, Furukawa M, Yoshizaki T (2009) Malignant phosphaturic mesenchymal tumor, mixed connective tissue variant of the tongue. Auris Nasus Larynx 36:104-105

187 Woo VL, Landesberg R, Imel EA, Singer SR, Folpe AL, Econs MJ, Kim T, Harik LR, Jacobs TP (2009) Phosphaturic mesenchymal tumor, mixed connective tissue variant, of the mandible: report of a case and review of the literature. Oral Surg Oral Med Oral Pathol Oral Radiol Endod 108:925-932

188 Yun KI, Kim DH, Pyo SW (2009) A phosphaturic mesenchymal tumor of the floor of the mouth with oncogenic osteomalacia: report of a case. J Oral Maxillofac Surg 67:402-405

189 Bee YM, Chan LP, Goh CHK (2010) A man with persistent, enigmatic bone pain and hypophosphatemia. Endocrinologist 20:33-37

190 Biernat W, Kaniuka S, Stempniewicz M, Reclawowicz D, Sworczak K (2010) Phosphaturic mesenchymal tumor of spinal nerve in a patient with osteomalacia and multiple fractures. Acta Neuropathologica 119:379-380

191 Chouhan V, Agrawal K, Vinothkumar TK, Mathesul A (2010) Bilateral insufficiency fracture of the femoral head and neck in a case of oncogenic osteomalacia. Journal of Bone and Joint Surgery - Series B 92:1028-1031

192 Fok AWM, Ng TP (2010) Osteomalacia: A case series of patients with atypical clinical orthopaedic presentations. Hong Kong Medical Journal 16:476-479

193 Haeusler G, Freilinger M, Dominkus M, Egerbacher M, Amann G, Kolb A, Schlegel W, Raimann A, Staudenherz A (2010) Tumor-induced hypophosphatemic rickets in an adolescent boy - Clinical presentation, diagnosis, and histological findings in growth plate and muscle tissue. Journal of Clinical Endocrinology and Metabolism 95:4511-4517

194 Ing SW, Iwenofu OH, Mayerson JL, Hall NC (2010) Tumor-induced Osteomalacia Localization by Whole-body Sestamibi Scan. Endocrinologist 20:267-270

195 Ishii A, Imanishi Y, Kobayashi K, Hashimoto J, Ueda T, Miyauchi A, Koyano HM, Kaji H, Saito T, Oba K, Komatsu Y, Kurajoh M, Nagata Y, Goto H, Wakasa K, Sugimoto T, Miki T, Inaba M, Nishizawa Y (2010) The levels of somatostatin receptors in causative tumors of oncogenic osteomalacia are insufficient for their agonist to normalize serum phosphate levels. Calcif Tissue Int 86:455-462

196 Ito N, Shimizu Y, Suzuki H, Saito T, Okamoto T, Hori M, Akahane M, Fukumoto S, Fujita T (2010) Clinical utility of systemic venous sampling of FGF23 for identifying tumours responsible for tumour-induced osteomalacia: Letter to the Editor. Journal of Internal Medicine 268:390-394

197 Jung GH, Kim JD, Cho Y, Chung SH, Lee JH, Sohn KR (2010) A 9-month-old phosphaturic mesenchymal tumor mimicking the intractable rickets. J Pediatr Orthop B 19:127-132

198 Kurien R, Manipadam MT, Rupa V (2010) Oncogenic osteomalacia in a patient with an ethmoid sinus tumour. J Laryngol Otol 124:799-803

199 Malhotra G, Agrawal A, Jambhekar NA, Sarathi V, Jagtap V, Agarwal MG, Kulkarni M, Asopa RV, Shah NS (2010) Interesting image. The search for primary tumor in a patient with oncogenic osteomalacia: F-18 FDG PET resolves the conundrum. Clin Nucl Med 35:896-898

200 Mori Y, Ogasawara T, Motoi T, Shimizu Y, Chikazu D, Tamura K, Fukumoto S, Takato T (2010) Tumor-induced osteomalacia associated with a maxillofacial tumor producing fibroblast growth factor 23: report of a case and review of the literature. Oral Surg Oral Med Oral Pathol Oral Radiol Endod 109:e57-63

201 Parshwanath HA, Kulkarni PR, Rao R, Joshi SK, Patil P (2010) Phosphaturic mesenchymal tumor of ethmoid sinus. Indian Journal of Pathology and Microbiology 53:384-385

202 Peters KB, McLendon R, Morse MA, Vredenburgh JJ (2010) Treatment of Recurrent Intracranial Hemangiopericytoma with SRC-Related Tyrosine Kinase Targeted Therapy: A Case Report. Case Rep Oncol 3:93-97

203 Quintana Duque MA, Varela Nariño A, Rondón F, Félix Restrepo J, Iglesias Gamarra A (2010) Tumor-induced osteomalacia. Revista Colombiana de Reumatologia 17:249-256

204 Rao J, Varaprasad IR, Rajasekhar L, Taduri G, Challa S, Gumdal N, Venkatratnam SV (2010) What is your diagnosis?: Unusual cause of osteomalacia. Indian Journal of Rheumatology 5:93–94

205 Vijay AP, Tan ATB, Suhaida AM, Chan SP (2010) Oncogenic osteomalacia, you say? Better start looking then-A Case Report. Journal of Health and Translational Medicine 13:63-68

206 Xia WB, Jiang Y, Li M, Xing XP, Wang O, Hu YY, Zhang HB, Liu HC, Meng XW, Zhou XY (2010) Levels and dynamic changes of serum fibroblast growth factor 23 in hypophosphatemic rickets/osteomalacia. Chin Med J (Engl) 123:1158-1162

207 Yan MT, Wu MJ, Hsaio PJ, Lin SH (2010) The case a 42-year-old male with 3-year bone pain and a soft tissue mass. Kidney International 78:823-824

208 Akhter M, Sugrue PA, Bains R, Khavkin YA (2011) Oncogenic osteomalacia of the cervical spine: a rare case of curative resection and reconstruction. J Neurosurg Spine 14:453-456

209 Andreopoulou P, Dumitrescu CE, Kelly MH, Brillante BA, Cutler Peck CM, Wodajo FM, Chang R, Collins MT (2011) Selective venous catheterization for the localization of phosphaturic mesenchymal tumors. Journal of Bone and Mineral Research 26:1295-1302

210 Bergwitz C, Collins MT, Kamath RS, Rosenberg AE (2011) Case 33-2011: A 56-year-old man with hypophosphatemia. New England Journal of Medicine 365:1625-1635

211 González DC, Peratta M (2011) An inusual clinical presentation of a tumor-induced osteomalacia. Bone 49:1378

212 Hendry DS, Wissman R (2011) Case 165: Oncogenic osteomalacia. Radiology 258:320-322

213 Hu FK, Yuan F, Jiang CY, Lv DW, Mao BB, Zhang Q, Yuan Z, Wang Y (2011) Tumor-induced osteomalacia with elevated fibroblast growth factor 23: C case phosphaturic mesenchymal tumor mixed with connective tissue variants and review of the literature. Chinese Journal of Cancer 30:794-804

214 Jagtap VS, Sarathi V, Lila AR, Malhotra G, Sankhe SS, Bandgar T, Menon P, Shah NS (2011) Tumor-induced osteomalacia: A single center experience. Endocrine Practice 17:177-184

215 Kaji H, Imanishi Y, Sugimoto T, Seino S (2011) Comparisons of serum sclerostin levels among patients with postmenopausal osteoporosis, primary hyperparathyroidism and osteomalacia. Experimental and Clinical Endocrinology and Diabetes 119:440-444

216 Kletter GB, Dales MC, Shorr SS, Boyce A, Chong WH, Wodajo F, Chang R, Chen C, Collins MT (2011) Utility of FGF23 venous sampling in a child with hypophosphatemic rickets. Endocrine Reviews 32

217 Kobayashi K, Nakao K, Kawai K, Ito K, Hukumoto S, Asakage T, Oota S, Motoi R (2011) Tumor-induced osteomalacia originating from the temporal bone: A case report. Head and Neck 33:1072-1075

218 Lam JKY, Lam CW, Kung AWC, Tan KCB, Lau KS, Lam KSL (2011) Case report A man with hypophosphataemia. British Medical Journal 342

219 Markou A, Tsiama V, Tournis S, Papanastasiou L, Tsiavos V, Dassou A, Vlachou V, Papaliodi E, Asimaki N, Zografos G, Piaditis G (2011) Coexistence of tumor-induced osteomalacia and primary hyperparathyroidism. Endocrine Practice 17:e144-e148

220 Ozawa JC, De Paiva AFN, De Próspero JD, Monte O, Scalissi NM, Maeda SS (2011) Mesenchymal tumor in the calcaneus with oncogenic osteomalacia: A case report. Journal of Clinical Oncology 29:e6-e8

221 Seo HJ, Choi YJ, Kim HJ, Jeong YH, Cho A, Lee JH, Yun M, Lee JD, Kang WJ (2011) Using 18F-FDG PET/CT to Detect an Occult Mesenchymal Tumor Causing Oncogenic Osteomalacia. Nuclear Medicine and Molecular Imaging 45:233-237

222 Suryawanshi P, Agarwal M, Dhake R, Desai S, Rekhi B, Reddy KB, Jambhekar NA (2011) Phosphaturic mesenchymal tumor with chondromyxoid fibroma-like feature: An unusual morphological appearance. Skeletal Radiology 40:1481-1485

223 Tantisattamo E, Ng RC (2011) Dual paraneoplastic syndromes: small cell lung carcinoma-related oncogenic osteomalacia, and syndrome of inappropriate antidiuretic hormone secretion: report of a case and review of the literature. Hawaii medical journal 70:139-143

224 Thompson T, Chong WH, Kelly MH, Andeopolis P, Collins MT, Banerji MA (2011) Tumor-induced osteomalacia (TIO): Treatment and course of recovery. Endocrine Reviews 32

225 Tournier A, Hanslik T, de la Faille R, Trad S, Baglin A, Prinseau J, Moulonguet-Doleris L (2011) [Oncogenic osteomalacia: increased production of fibroblast growth factor 23 is not the unique actor] Osteomalacie oncogenique : le FGF 23 ne fait pas tout. Rev Med Interne 32:e99-e101

226 Uno T, Kawai K, Kunii N, Fukumoto S, Shibahara J, Motoi T, Saito N (2011) Osteomalacia caused by skull base tumors: Report of 2 cases. Neurosurgery 69:E239-E244

227 Westerberg PA, Linde T, Eklof H, Ljunggren O (2011) REPEATED VENOUS SAMPLING FOR DETERMINATION OF A GRADIENT OF FIBROBLAST GROWTH FACTOR 23 FOR LOCALIZATION OF AN OSTEOMALACIA CAUSING TUMOR. Acta Endocrinologica-Bucharest 7:395-403

228 Zornizki T, Schattner A, Coslovsky R, Collins MT (2011) Height loss and generalized pain in a 35-year-old man. Am J Med 124:e3-5

229 Battoo AJ, Salih S, Unnikrishnan AG, Jojo A, Bahadur S, Iyer S, Kuriakose MA (2012) Oncogenic osteomalacia from nasal cavity giant cell tumor. Head and Neck 34:454-457

230 Brandwein-Gensler M, Siegal GP (2012) Striking Pathology Gold: A Singular Experience with Daily Reverberations: Sinonasal Hemangiopericytoma (Glomangiopericytoma) and Oncogenic Osteomalacia. Head and Neck Pathology 6:64-74

231 Burnand H, Samuels A, Hagan I, Sawant N, Mutimer J (2012) Bilateral subtrochanteric fractures in tumour-induced osteomalacia caused by a nasal haemangiopericytoma. Hip International 22:227-229

232 Chang CV, Conde SJ, Luvizotto RAM, Nunes VS, Bonates MC, Felicio AC, Lindsey SC, Moraes FH, Tagliarini JV, Mazeto GMFS, Kopp P, Nogueira CR (2012) Oncogenic osteomalacia: Loss of hypophosphatemia might be the key to avoid misdiagnosis. Arquivos Brasileiros de Endocrinologia e Metabologia 56:570-573

233 Cho SI, Do NY, Yu SW, Choi JY (2012) Nasal hemangiopericytoma causing oncogenic osteomalacia. Clin Exp Otorhinolaryngol 5:173-176

234 Chokyu I, Ishibashi K, Goto T, Ohata K (2012) Oncogenic osteomalacia associated with mesenchymal tumor in the middle cranial fossa: A case report. Journal of Medical Case Reports 6

235 Drummond JB, Soares MMS, Dias RP, Dias Filho MA (2012) Oncogenic osteomalacia: Case report. Archives of Osteoporosis 7:S190-S191

236 Elfenbein DM, Weber TJ, Scheri RP (2012) Tumor-induced osteomalacia masking primary hyperparathyroidism. Surgery (United States) 152:1256-1258

237 Jiang Y, Xia WB, Xing XP, Silva BC, Li M, Wang O, Zhang HB, Li F, Jing HL, Zhong DR, Jin J, Gao P, Zhou L, Qi F, Yu W, Bilezikian JP, Meng XW (2012) Tumor-induced osteomalacia: An important cause of adult-onset hypophosphatemic osteomalacia in China: Report of 39 cases and review of the literature. Journal of Bone and Mineral Research 27:1967-1975

238 Kaniuka-Jakubowska S, Biernat W, Lewczuk A, Świa̧tkowska-Stodulska R, Sworczak K (2012) Oncogenic osteomalacia should be considered in hypophosphatemia, bone pain and pathological fractures. Endokrynologia Polska 63:234-238

239 Mak MP, Da Costa e Silva VT, Martin RM, Lerario AM, Yu L, Hoff PMG, De Castro Junior G (2012) Advanced prostate cancer as a cause of oncogenic osteomalacia: An underdiagnosed condition. Supportive Care in Cancer 20:2195-2197

240 Mendoza-Ramírez S, Gutiérrez-Mijangos O, Sánchez-Silva AC, De Jesús Navarro-Córdoba F, Murguía-Pérez M (2012) Phosphaturic mesenchymal tumour with giant rosettes: A rare cause of oncogenic osteomalacia. Revista Espanola de Patologia 45:53-57

241 Munoz J, Ortega RM, Celzo F, Donthireddy V (2012) Tumour-induced osteomalacia. BMJ Case Reports

242 Pandey R, Agarwal A, Darlong V, Garg R, Punj J (2012) Perioperative concerns in patients with tumorinduced osteomalacia for surgical excision of tumor. Annals of Saudi Medicine 32:656-658

243 Ramachandran R, Rewari V, Trikha A, Singh PM (2012) Anesthesia for oncogenic osteomalacia--a rare paraneoplastic syndrome. Acta Anaesthesiol Taiwan 50:134-137

244 Tutton S, Olson E, King D, Shaker JL (2012) Successful treatment of tumor-induced osteomalacia with CT-guided percutaneous ethanol and cryoablation. Journal of Clinical Endocrinology and Metabolism 97:3421-3425

245 Wang XL, Ba JM, Zhong WW, Lü ZH, Dou JT, Lu JM, Mu YM (2012) Osteomalacia caused by tumors in facies cranii mimicking rheumatoid arthritis. Rheumatology International 32:2573-2576

246 Westerberg PA, Linde T, Vanderschueren D, Billen J, Jans I, Ljunggren O (2012) Oncogenic osteomalacia illustrating the effect of fibroblast growth factor 23 on phosphate homeostasis. CKJ: Clinical Kidney Journal 5:240-243

247 William J, Laskin W, Nayar R, De Frias D (2012) Diagnosis of phosphaturic mesenchymal tumor (mixed connective tissue type) by cytopathology. Diagnostic Cytopathology 40:E109-E113

248 Andzel G, Terry TL, Shaik S, Mellati M, Plummer EV (2013) The little-big troublemaker-A case of oncogenic hypophosphatemic osteomalacia leading to irreversible renal failure and fragility bone fractures. Endocrine Reviews 34

249 Belachew D, Andrews RG, Baumhardt HI, Sperling MA (2013) Oral brown tumors in familial hypophosphatemic rickets. Endocrine Reviews 34

250 Chiam P, Tan HC, Bee YM, Chandran M (2013) Oncogenic osteomalacia - Hypophosphataemic spectrum from "benignancy" to "malignancy". Bone 53:182-187

251 Chong WH, Andreopoulou P, Chen CC, Reynolds J, Guthrie L, Kelly M, Gafni RI, Bhattacharyya N, Boyce AM, El-Maouche D, Crespo DO, Sherry R, Chang R, Wodajo FM, Kletter GB, Dwyer A, Collins MT (2013) Tumor localization and biochemical response to cure in tumor-induced osteomalacia. Journal of Bone and Mineral Research 28:1386-1398

252 Clifton-Bligh RJ, Hofman MS, Duncan E, Sim IW, Darnell D, Clarkson A, Wong T, Walsh JP, Gill AJ, Ebeling PR, Hicks RJ (2013) Improving diagnosis of tumor-induced osteomalacia with gallium-68 DOTATATE PET/CT. Journal of Clinical Endocrinology and Metabolism 98:687-694

253 de Jongh RT, Vervloet MG, Bravenboer N, Heijboer AC, den Heijer M, Lips P (2013) [Chronic bone pain due to raised FGF23 production? The importance of determining phosphate levels] Chronische botpijn door verhoogde FGF23-productie? Het belang van een fosfaatbepaling. Ned Tijdschr Geneeskd 157:A5908

254 Gardner KH, Shon W, Folpe AL, Wieland CN, Tebben PJ, Baum CL (2013) Tumor-induced osteomalacia resulting from primary cutaneous phosphaturic mesenchymal tumor: a case and review of the medical literature. Journal of cutaneous pathology 40:780-784; quiz 779

255 Jin X, Jing H, Li F, Zhuang H (2013) Osteomalacia-inducing renal clear cell carcinoma uncovered by 99mTc-Hydrazinonicotinyl-Tyr3-Octreotide (99mTc-HYNIC-TOC) scintigraphy. Clinical Nuclear Medicine 38:922-924

256 Jing H, Li F, Zhong D, Zhuang H (2013) 99mTc-HYNIC-TOC (99mTc-hydrazinonicotinyl-Tyr3- octreotide) scintigraphy identifying two separate causative tumors in a patient with tumor-induced osteomalacia (TIO). Clinical Nuclear Medicine 38:664-667

257 Koehne T, Marshall RP, Jeschke A, Kahl-Nieke B, Schinke T, Amling M (2013) Osteopetrosis, osteopetrorickets and hypophosphatemic rickets differentially affect dentin and enamel mineralization. Bone 53:25-33

258 Leaf DE, Pereira RC, Bazari H, Jüppner H (2013) Oncogenic osteomalacia due to FGF23-expressing colon adenocarcinoma. Journal of Clinical Endocrinology and Metabolism 98:887-891

259 Ledford CK, Zelenski NA, Cardona DM, Brigman BE, Eward WC (2013) The phosphaturic mesenchymal tumor: Why is definitive diagnosis and curative surgery often delayed? Clinical Orthopaedics and Related Research 471:3618-3625

260 Manzil FFP, Bhambhvani PG, O'Malley JP (2013) Evaluation of tumor-induced osteomalacia with 111in-pentetreotide scintigraphy. Journal of Nuclear Medicine Technology 41:299-301

261 Mathis DA, Stehel Jr EJ, Beshay JE, Mickey BE, Folpe AL, Raisanen J (2013) Intracranial phosphaturic mesenchymal tumors: Report of 2 cases. Journal of Neurosurgery 118:903-907

262 Nakanishi K, Sakai M, Tanaka H, Tsuboi H, Hashimoto J, Hashimoto N, Tomiyama N (2013) Whole-body MR imaging in detecting phosphaturic mesenchymal tumor (PMT) in tumor-induced hypophosphatemic osteomalacia. Magnetic Resonance in Medical Sciences 12:47-52

263 Nanes MS (2013) Phosphate wasting and fibroblast growth factor-23. Current Opinion in Endocrinology, Diabetes and Obesity 20:523-531

264 Niemeier T, Leddy L, Bolster M, Chapin R (2013) Insufficiency fracture associated with oncogenic osteomalacia. Journal of Clinical Rheumatology 19:38-42

265 Papierska L, Ćwikła JB, Misiorowski W, Rabijewski M, Sikora K, Wanyura H (2013) Unusual case of phosphaturic mesenchymal tumor. Polskie Archiwum Medycyny Wewnetrznej 123:255-256

266 Puthenveetil PJ, Hattab EM, Peacock M, Horn EM (2013) Thoracic phosphaturic mesenchymal tumors causing oncogenic osteomalacia. Journal of Clinical Neuroscience 20:1057-1061

267 Sánchez A, Castiglioni A, Cóccaro N, Silva R, Bobrovsky E, Moysés RMA, Graciolli F (2013) Ostemalacia due to a tumor secreting FGF-23. Medicina (Argentina) 73:43-46

268 Serafini EM, Pisarevsky AA, Garrido JP, Zamora RJ, Petrucci EA (2013) Tumor-induced osteomalacia: Rhinosinusal hemangiopericytoma. Medicina (Argentina) 73:39-42

269 Tarasova VD, Trepp-Carrasco AG, Thompson R, Recker RR, Chong WH, Collins MT, Armas LAG (2013) Successful treatment of tumor-induced osteomalacia due to an intracranial tumor by fractionated stereotactic radiotherapy. Journal of Clinical Endocrinology and Metabolism 98:4267-4272

270 Xie Y, Li HZ (2013) Oncogenic osteomalacia caused by renal cell carcinoma. Journal of Clinical Endocrinology and Metabolism 98:4597-4598

271 Yasuda S, Wada S, Kono S, Miyajima T, Oda H, Katayama S, Awata T (2013) Tumor-induced osteomalacia: Benign tumor recurrence after two surgical resections at two different medical institutions. Endocrine Practice 19:e97-e101

272 Amblee A, Uy J, Senseng C, Hart P (2014) Tumor-induced osteomalacia with normal systemic fibroblast growth factor-23 level. Clinical Kidney Journal 7:186-189

273 Bhatt AA, Mathews SS, Kumari A, Paul TV (2014) Tumour-induced osteomalacia. Hong Kong Medical Journal 20:350.e351-350.e352

274 Dai-Ju JQ, Surampudi V, Jirajariyavej T, Gianoukakis AG (2014) Severe hypophosphatemia due to a FGF-23 producing, metastatic, aggressive tumor of unknown primary. Endocrine Reviews 35

275 Garcha A, Berns J, Kobrin S, Goldfarb S (2014) Oncogenic osteomalacia (OO): Renal phosphate wasting associated with metastatic prostate cancer. American Journal of Kidney Diseases 63:A48

276 Gulwani H, Garg N (2014) A 40-year-old woman with intracranial bleed and osteomalacia. Brain Pathology 24:419-420

277 Hautmann AH, Schroeder J, Wild P, Hautmann MG, Huber E, Hoffstetter P, Fleck M, Girlich C, Capella C (2014) Tumor-induced osteomalacia: Increased level of FGF-23 in a patient with a phosphaturic mesenchymal tumor at the tibia expressing periostin. Case Reports in Endocrinology 2014

278 Hu S, Lubitz S (2014) Tumor-induced osteomalacia caused by a FGF-23 producing mesenchymal tumor. Journal of General Internal Medicine 29:S460

279 Iguchi G, Matsumoto R, Bando H, Suda K, Nishizawa H, Takahashi M, Fukuoka H, Imanishi Y, Sasano H, Takahashi Y (2014) A case of androgen-and fibroblast growth factor 23-producing ovarian tumor. Endocrine Reviews 35

280 Jadhav S, Kasaliwal R, Lele V, Rangarajan V, Chandra P, Shah H, Malhotra G, Jagtap VS, Budyal S, Lila AR, Bandgar T, Shah NS (2014) Functional imaging in primary tumour-induced osteomalacia: Relative performance of FDG PET/CT vs somatostatin receptor-based functional scans: A series of nine patients. Clinical Endocrinology 81:31-37

281 Jadhav S, Kasaliwal R, Shetty NS, Kulkarni S, Rathod K, Popat B, Kakade H, Bukan A, Khare S, Budyal S, Jagtap VS, Lila AR, Bandgar T, Shah NS (2014) Radiofrequency ablation, an effective modality of treatment in tumor-induced osteomalacia: A case series of three patients. Journal of Clinical Endocrinology and Metabolism 99:3049-3054

282 Kim Y, Stein E, Remotti F, Lee FY (2014) Tumor-induced osteomalacia secondary to a fibroblast growth factor 23-secreting phosphaturic mesenchymal tumor in the foot. JBJS Case Connector 4

283 Koplas MC, Rubin BP, Sundaram M (2014) Phosphaturic mesenchymal tumor: Two contrasting cases. Skeletal Radiology 43:841-845

284 Latifyan SB, Vanhaeverbeek M, Klastersky J (2014) Tumour-associated osteomalacia and hypoglycaemia in a patient with prostate cancer: Is Klotho involved? BMJ Case Reports 2014

285 Lee GG, Dhong HJ, Park YS, Ko YH (2014) Sinonasal glomangiopericytoma causing oncogenic osteomalacia. Clin Exp Otorhinolaryngol 7:145-148

286 Leow MKS, Hamijoyo L, Liew H, Thirugnanam U, Cheng MHW, Loke KSH, Teo MSK, Chuah KL, Chng HH (2014) Oncogenic osteomalacia presenting as a crippling illness in a young man. The Lancet 384:1236

287 Lin HA, Shih SR, Tseng YT, Chen CH, Chiu WY, Hsu CY, Tsai KS (2014) Ovarian cancer-related hypophosphatemic osteomalacia--a case report. J Clin Endocrinol Metab 99:4403-4407

288 Meng QH, Wagar EA (2014) Severe Hypophosphatemia in a 79-Year-Old Man. Clinical Chemistry 60:928-931

289 Monappa V, Naik AM, Mathew M, Rao L, Rao SK, Ramachandra L, Padmapriya J (2014) Phosphaturic mesenchymal tumour of the mandible - the useful criteria for a diagnosis on fine needle aspiration cytology. Cytopathology 25:54-56

290 Morimoto T, Takenaka S, Hashimoto N, Araki N, Myoui A, Yoshikawa H (2014) Malignant phosphaturic mesenchymal tumor of the pelvis: A report of two cases. Oncology Letters 8:67-71

291 Morrell NT, Beck NL, Clericuzio C, Frias-Kletecka C, Szalay EA (2014) Tumor-induced rickets presenting in an adolescent: A case report and review of the literature. JBJS Case Connector 4

292 Norden AGW, Laing RJC, Rowe P, Unwin RJ, Wrong O, Crisp AJ (2014) Oncogenic osteomalacia, raised FGF-23, and renal fanconi syndrome. QJM 107:139-141

293 Okubo Y, Arakaki O, Yamaguchi S, Uezato H, Takahashi K (2014) A case of tumor-induced osteomalacia. Journal of Dermatology 41:89

294 Piemonte S, Romagnoli E, Cipriani C, De Lucia F, Pilotto R, Diacinti D, Pepe J, Minisola S (2014) Six-year follow-up of a characteristic osteolytic lesion in a patient with tumor-induced osteomalacia. European Journal of Endocrinology 170:K1-K4

295 Pisani D, Todde F, Marafini J, Argento G, Bartolazzi A (2014) A case of hypophosphatemia sustained by ectopic secretion of FGF23. Italian Journal of Medicine 8:106

296 Pithankuakul K, Ratanasuwan T, Thanakit V, Sukhantanak B, Kiatisevi P (2014) Oncogenic osteomalacia caused by phosphaturic mesenchymal tumours in the proximal and shaft of the tibia: a case report. Journal of orthopaedic surgery (Hong Kong) 22:257-262

297 Postlethwaite C, Kumar J (2014) An aggressive bone tumour: Osteogenic osteomalacia. Clinical Medicine, Journal of the Royal College of Physicians of London 14:90

298 Reddy HB, Mounika G, Sri Nagesh V, Rao IS (2014) An interesting case of reversible metabolic myopathy: Tumor induced osteomalacia. Annals of Indian Academy of Neurology 17:237-238

299 Sahoo J, Balachandran K, Kamalanathan S, Das AK, Patro DK, Halanaik D, Badhe B (2014) Tumor(s) induced osteomalacia--a curious case of double trouble. J Clin Endocrinol Metab 99:395-398

300 Sinnakirouchenan R, Trivedi HS (2014) Hypophosphatemia secondary to tumor induced osteomalacia in metastatic prostate cancer. American Journal of Kidney Diseases 63:A104

301 Wahner-Roedler DL (2014) Oncogenic osteomalacia: A challenging diagnosis. Journal of General Internal Medicine 29:S399

302 Angeles-Angeles A, Reza-Albarrán A, Chable-Montero F, Cordova-Ramón JC, Albores-Saavedra J, Martinez-Benitez B (2015) Phosphaturic mesenchymal tumors. Survey of 8 cases from a single Mexican medical institution. Annals of Diagnostic Pathology 19:375-380

303 Burckhardt MA, Schifferli A, Krieg AH, Baumhoer D, Szinnai G, Rudin C (2015) Tumor-associated FGF-23-induced hypophosphatemic rickets in children: a case report and review of the literature. Pediatric Nephrology 30:179-182

304 Farmakis SG, Siegel MJ (2015) Phosphaturic mesenchymal tumor of the tibia with oncogenic osteomalacia in a teenager. Pediatric Radiology 45:1423-1426

305 Fathalla H, Cusimano M, Di Ieva A, Karamchandani J, Fung R, Kovacs K (2015) Osteomalacia-Inducing Tumors of the Brain: A Case Report, Review and a Hypothesis. World Neurosurgery 84:189.e181-189.e185

306 Fernández-Cooke E, Cruz-Rojo J, Gallego C, Romance AI, Mosqueda-Peña R, Almaden Y, Sánchez Del Pozo J (2015) Tumor-induced rickets in a child with a central giant cell granuloma: A case report. Pediatrics 135:e1518-e1523

307 Higley M, Beckett B, Schmahmann S, Dacey E, Foss E (2015) Locally aggressive and multifocal phosphaturic mesenchymal tumors: two unusual cases of tumor-induced osteomalacia. Skeletal Radiology 44:1825-1831

308 Ho CL (2015) Ga68-DOTA Peptide PET/CT to Detect Occult Mesenchymal Tumor-Inducing Osteomalacia: A Case Series of Three Patients. Nuclear Medicine and Molecular Imaging 49:231-236

309 Jerkovich F, Moncet D, Babini S, Zoppi JA, Graciolli F, Oliveri B (2015) [Oncogenic osteomalacia. Report of two cases]

Osteomalacia oncogenica. Presentacion de dos casos. Medicina (B Aires) 75:37-40

310 Kumar R, Folpe AL, Mullan BP (2015) Tumor-Induced Osteomalacia. Transl Endocrinol Metab 7

311 Masood MQ, Ram N, Ali SA (2015) Tumour induced osteomalacia. Journal of the Pakistan Medical Association 65:220-222

312 Meng T, Zhou W, Li B, Yin H, Li Z, Zhou L, Kong J, Yan W, Yang X, Liu T, Song D, Xiao J (2015) En bloc resection for treatment of tumor-induced osteomalacia: A case presentation and a systematic review. World Journal of Surgical Oncology 13

313 Mittal M, Jain N, Wakhlu A, Gambhir S (2015) Oncogenic osteomalacia caused by multiple soft tissue tumors. Endocrine Reviews 36

314 Nagae K, Uchi H, Ito T, Moroi Y, Oda Y, Furue M (2015) Osteomalacia induced by a phosphaturic mesenchymal tumor secreting fibroblast growth factor 23. European Journal of Dermatology 25:199-200

315 Nakamura T, Aizawa T, Hoshikawa T, Ozawa H, Ito N, Fukumoto S, Itoi E, Kokubun S (2015) Tumor-induced osteomalacia caused by phosphaturic mesenchymal tumor of the cervical spine. Journal of Orthopaedic Science 20:765-771

316 Nakhoul NF, El-Hajj Fuleihan G, Haidar M, Shoucair M, Hourani M, Arabi A (2015) Tumor induced osteomalacia associated with a giant cell tumor mimicking inguinal lymphnode, a lesson to learn from each case. Endocrine Reviews 36

317 Okamiya T, Takahashi K, Kamada H, Hirato J, Motoi T, Fukumoto S, Chikamatsu K (2015) Oncogenic osteomalacia caused by an occult paranasal sinus tumor. Auris Nasus Larynx 42:167-169

318 Pallavi R, Ravella PM, Gupta P, Popescu A (2015) A Case of Phosphaturic Mesenchymal Tumor. American Journal of Therapeutics 22:e57-e61

319 Ray S, Chakraborty PP, Biswas K, Beatrice AM, Ghosh S, Mukhopadhyay S, Chowdhury S (2015) Oncogenic osteomalacia caused by occult nasal mesenchymal tumor: A monster in the cave. Oxford Medical Case Reports 2015:265-268

320 Shetty S, Kapoor N, Cherian VM, Paul TV (2015) An unusual treatable cause for proximal muscle weakness. BMJ Case Reports 2015

321 Shustik DA, Ng DC, Sittampalam K (2015) Phosphaturic mesenchymal tumour mixed connective tissue variant: report of three cases with unusual histological findings. International journal of clinical and experimental pathology 8:7506-7517

322 Sun ZJ, Jin J, Qiu GX, Gao P, Liu Y (2015) Surgical treatment of tumor-induced osteomalacia: A retrospective review of 40 cases with extremity tumors. BMC Musculoskeletal Disorders 16

323 Tang EY, Sowden E (2015) A rare case of tumour induced osteomalacia in rheumatology. Rheumatology (United Kingdom) 54:i57

324 Wang H, Zhong D, Liu Y, Jiang Y, Qiu G, Weng X, Xing X, Li M, Meng X, Li F, Zhu Z, Yu W, Xia W, Jin J (2015) Surgical treatments of tumor-induced osteomalacia lesions in long bones seventeen cases with more than one year of follow-up. Journal of Bone and Joint Surgery - American Volume 97:1084-1094

325 Yavropoulou MP, Gerothanasi N, Frydas A, Triantafyllou E, Poulios C, Hytiroglou P, Apostolou P, Papasotiriou I, Tournis S, Kesisoglou I, Yovos JG (2015) Tumor-induced osteomalacia due to a recurrent mesenchymal tumor overexpressing several growth factor receptors. Endocrinology, Diabetes and Metabolism Case Reports 2015

326 Zhang J, Zhu Z, Zhong D, Dang Y, Xing H, Du Y, Jing H, Qiao Z, Xing X, Zhuang H, Li F (2015) 68Ga DOTATATE PET/CT is an Accurate Imaging Modality in the Detection of Culprit Tumors Causing Osteomalacia. Clin Nucl Med 40:642-646

327 Abate EG, Bernet V, Cortese C, Garner HW (2016) Tumor induced osteomalacia secondary to anaplastic thyroid carcinoma: A case report and review of the literature. Bone Reports 5:81-85

328 Alonso G, Varsavsky M (2016) Osteomalacia in a young adult. Revista de Osteoporosis y Metabolismo Mineral 8:82-86

329 Annamalai AK, Sampathkumar K, Kane S, Shetty NS, Kulkarni S, Rangarajan V, Purandare N, Pai PS, Mahuvakar AD, Shanthi R, Suriyakumar G, Puri V, Aram S, Gopalakrishnan C, Chelian M, Srinivasan KG, Gill AJ, Gurnell M, Clifton-Bligh R (2016) Needle(s) in the haystack-synchronous multifocal tumor-induced osteomalacia. Journal of Clinical Endocrinology and Metabolism 101:390-393

330 Basu S, Fargose P (2016) 177Lu-DOTATATE PRRT in recurrent skull-base phosphaturic mesenchymal tumor causing osteomalacia: A potential application of PRRT beyond neuroendocrine tumors. Journal of Nuclear Medicine Technology 44:248-250

331 Bhavani N, Reena Asirvatham A, Kallur K, Menon AS, Pavithran PV, Nair V, Vasukutty JR, Menon U, Kumar H (2016) Utility of Gallium-68 DOTANOC PET/CT in the localization of Tumour-induced osteomalacia. Clinical Endocrinology 84:134-140

332 Dadoniene J, Miglinas M, Miltiniene D, Vajauskas D, Seinin D, Butenas P, Kacergius T (2016) Tumour-induced osteomalacia: A literature review and a case report. World Journal of Surgical Oncology 14

333 Dhir V, Sagar V, Bhadada S, Prakash M, Radotra BD, Rattan V (2016) Bony pains originating from a tooth! Joint Bone Spine 83:225

334 Elderman JH, Wabbijn M, De Jongh F (2016) Hypophosphataemia due to FGF-23 producing B cell non-Hodgkin's lymphoma. BMJ Case Reports 2016

335 Hu F, Jiang C, Zhang Q, Shi H, Wei L, Wang Y (2016) Quantitative ELISA-Like Immunohistochemistry of Fibroblast Growth Factor 23 in Diagnosis of Tumor-Induced Osteomalacia and Clinical Characteristics of the Disease. Disease Markers 2016

336 Jain A, Shelley S, Muthukrishnan I, Kalal S, Amalachandran J, Chandran S (2016) Diagnostic importance of contrast enhanced 18 F-fluorodeoxyglucose positron emission computed tomography in patients with tumor induced osteomalacia: Our experience. Indian Journal of Nuclear Medicine 31:14-19

337 Kang LE, Javanmard P, Kapoor A (2016) Tumor-induced osteomalacia in the setting of normal FGF-23. Endocrine Reviews 37

338 Karaa-Zbidi N, Damade R, Lhote F (2016) Inability to walk in a 41-year-old man. Revue De Medecine Interne 37:62-65

339 Kim I, Rajani R (2016) Oncogenic Osteomalacia From a Primary Phosphaturic Mesenchymal Tumor of the Toe: A Case Report. Journal of Foot and Ankle Surgery 55:294-298

340 Lokočová E, Cibiček N, Horák P, Flodrová P, Zadražil J (2016) Tumor-induced hypophosphatemia with osteomalacia - A case report. Osteologicky Bulletin 21:136-141

341 Maehara J, Yamashita K, Hiwatashi A, Togao O, Kikuchi K, Matsumoto Y, Iura K, Oda Y, Ichino I, Nakamoto Y, Honda H (2016) Primary phosphaturic mesenchymal tumour of the lumbar spine: utility of Ga-68-DOTATOC PET/CT findings. Bjr Case Reports 2

342 Maybody M, Grewal RK, Healey JH, Antonescu CR, Fanchon L, Hwang S, Carrasquillo JA, Kirov A, Farooki A (2016) Ga-68 DOTATOC PET/CT-Guided Biopsy and Cryoablation with Autoradiography of Biopsy Specimen for Treatment of Tumor-Induced Osteomalacia. CardioVascular and Interventional Radiology 39:1352-1357

343 Mok Y, Lee JC, Lum JHY, Petersson F (2016) From epistaxis to bone pain-report of two cases illustrating the clinicopathological spectrum of phosphaturic mesenchymal tumour with fibroblast growth factor receptor 1 immunohistochemical and cytogenetic analyses. Histopathology 68:925-930

344 Okiror L, Khalil H, Vaiyapuri S, Kalkat M (2016) Complete resection of a large phosphaturic mesenchymal tumour by chest wall resection and reconstruction. General Thoracic and Cardiovascular Surgery 64:355-358

345 Qari H, Hamao-Sakamoto A, Fuselier C, Cheng YSL, Kessler H, Wright J (2016) Phosphaturic Mesenchymal Tumor: 2 New Oral Cases and Review of 53 Cases in the Head and Neck. Head and neck pathology 10:192-200

346 Rodriguez-Velver KV, Zapata-Rivera MA, Montes-Villarreal J, Lavalle-González FJ, González-González JG, Villarreal-Pérez JZ, Rodríguez-Gutierrez R (2016) Tumour-induced osteomalacia secondary to a sarcoma. European Endocrinology 12:104-106

347 Sauder A, Wiernek S, Dai X, Pereira R, Yudd M, Patel C, Golden A, Ahmed S, Choe J, Chang V, Sender S, Cai D (2016) FGF23-Associated Tumor-Induced Osteomalacia in a Patient with Small Cell Carcinoma: A Case Report and Regulatory Mechanism Study. International Journal of Surgical Pathology 24:116-120

348 Slot-Steenks MMB, Hamdy NAT, van de Sande MAJ, Vriens D, Cleven AHG, Appelman-Dijkstra NM (2016) Identifying the culprit lesion in tumor induced hypophosphatemia, the solution of a clinical enigma. Endocrine 54:642-647

349 Wesorick DH, Brotman DJ, Jaffe C, Berg A, Chan MP, Houchens N (2016) Painful losses. Journal of Hospital Medicine 11:730-734

350 Wong B, Phan P, Santos M, Liu DM (2016) Tumour-induced osteomalacia of the cervical spine: A rare but important cause of hypophosphatemia. Endocrine Reviews 37

351 Akbari M, Larijani B, Sharghi S, Jalili A, Sajjadi-Jazi SM (2017) Is there any link between tumor-induced osteomalacia and psoriasis? A case report. Journal of Diabetes and Metabolic Disorders 16

352 Arai R, Onodera T, Terkawi MA, Mitsuhashi T, Kondo E, Iwasaki N (2017) A rare case of multiple phosphaturic mesenchymal tumors along a tendon sheath inducing osteomalacia. BMC Musculoskeletal Disorders 18

353 Beygi S, Denio A, Sharma TS (2017) The Foot That Broke Both Hips: A Case Report and Literature Review of Tumor-Induced Osteomalacia. Case Rep Rheumatol 2017:3191673

354 Chanukya GV, Mengade M, Goud J, Rao IS, Jain A (2017) Tumor-induced Osteomalacia: A Sherlock Holmes Approach to Diagnosis and Management. Ann Maxillofac Surg 7:143-147

355 Chazal T, Khanine V, Lidove O, Godot S, Ziza JM (2017) [Tumor-induced osteomalacia caused by a late-revealing phosphaturic mesenchymal tumor]

Osteomalacie secondaire a une tumeur mesenchymateuse phosphaturique de revelation tardive. Rev Med Interne 38:412-415

356 Cowan S, Lozano-Calderon SA, Uppot RN, Sajed D, Huang AJ (2017) Successful CT guided cryoablation of phosphaturic mesenchymal tumor in the soft tissues causing tumor-induced osteomalacia: a case report. Skeletal Radiology 46:273-277

357 Crossen SS, Zambrano E, Newman B, Bernstein JA, Messner AH, Bachrach LK, Twist CJ (2017) Tumor-induced osteomalacia in a 3-year-old with unresectable central giant cell lesions. Journal of Pediatric Hematology/Oncology 39:e21-e24

358 Dehghani M, Dabbaghmanesh MH, Khalafi-Nezhad A, Riazmontazer N, Dehghanian A, Vojdani R, Sasani M (2017) Glomus tumor as a cause of oncogenic osteomalacia. Clinical Cases in Mineral and Bone Metabolism 14:359-362

359 Dey B, Gochhait D, Subramanian H, Ponnusamy M (2017) Oncogenic osteomalacia: An approach to diagnosis with a case report. Journal of Clinical and Diagnostic Research 11:ED05-ED07

360 Florenzano P, Gafni RI, Collins MT (2017) Tumor-induced osteomalacia. Bone Reports 7:90-97

361 Franco DL, Thomas L (2017) Small cell lung cancer associated with multiple paraneoplastic syndromes. Biomedica 37:8-10

362 Gatti AP, Tonello L, Neto JAD, Teixeira UF, Goldoni MB, Fontes PRO, Sampaio JA, Lima LMP, Waechter FL (2017) Oncogenic hypophosphatemic osteomalacia: From the first signal of disease to the first signal of healthy. International Journal of Surgery Case Reports 30:130-133

363 Ghorbani-Aghbolaghi A, Darrow MA, Wang T (2017) Phosphaturic mesenchymal tumor (PMT): exceptionally rare disease, yet crucial not to miss. Autops Case Rep 7:32-37

364 González G, Baudrand R, Sepúlveda MF, Vucetich N, Guarda FJ, Villanueva P, Contreras O, Villa A, Salech F, Toro L, Michea L, Florenzano P (2017) Tumor-induced osteomalacia: experience from a South American academic center. Osteoporosis International 28:2187-2193

365 Green D, Mohorianu I, Piec I, Turner J, Beadsmoore C, Toms A, Ball R, Nolan J, McNamara I, Dalmay T, Fraser WD (2017) MicroRNA expression in a phosphaturic mesenchymal tumour. Bone Rep 7:63-69

366 Gresham MS, Shen S, Zhang YJ, Gallagher K (2017) Anterior Skull Base Glomangioma-Induced Osteomalacia. J Neurol Surg Rep 78:e9-e11

367 Hana T, Tanaka S, Nakatomi H, Shojima M, Fukumoto S, Ikemura M, Saito N (2017) Definitive surgical treatment of osteomalacia induced by skull base tumor and determination of the half-life of serum fibroblast growth factor 23. Endocrine Journal 64:1033-1039

368 Huynh KV (2017) A case report: Tumor-induced osteomalacia caused by elevated FGF23 secretion. International Journal of Rheumatic Diseases 20:85

369 Kawai S, Ariyasu H, Furukawa Y, Yamamoto R, Uraki S, Takeshima K, Warigaya K, Nakamoto Y, Akamizu T (2017) Effective localization in tumor-induced osteomalacia using68 Ga-DOTATOC-PET/CT, venous sampling and 3T-MRI. Endocrinology, Diabetes and Metabolism Case Reports 2017

370 Kobayashi H, Akiyama T, Okuma T, Shinoda Y, Oka H, Ito N, Fukumoto S, Tanaka S, Kawano H (2017) Three-dimensional fluoroscopic navigation-assisted surgery for tumors in patients with tumor-induced osteomalacia in the bones. Computer assisted surgery (Abingdon, England) 22:14-19

371 Lee JY, Park HS, Han S, Lim JK, Hong N, Park SI, Rhee Y (2017) Localization of oncogenic osteomalacia by systemic venous sampling of fibroblast growth factor 23. Yonsei Medical Journal 58:981-987

372 Mani MK, Panigrahi MK (2017) Unusual calvarial tumour-oncogenic osteomalacia. British Journal of Neurosurgery 31:495-496

373 Nair A, Chakraborty S, Dharmshaktu P, Tandon N, Gupta Y, Khadgawat R, Jabbar PK, Bal CS, Agarwal S, Ganie MA (2017) Peptide receptor radionuclide and octreotide: A novel approach for metastatic tumor-induced osteomalacia. Journal of the Endocrine Society 1:726-730

374 Qiu S, Cao LL, Qiu Y, Yan P, Li ZX, Du J, Sun LM, Zhang QF (2017) Malignant phosphaturic mesenchymal tumor with pulmonary metastasis. Medicine (United States) 96

375 Sandoval MAS, Palermo MA, Carrillo R, Bundoc R, Carnate JM, Galsim RJ (2017) Successful treatment of tumour-induced osteomalacia after resection of an oral peripheral ossifying fibroma. BMJ Case Reports 2017

376 Satyaraddi A, Cherian KE, Shetty S, Kapoor N, Jebasingh FK, Cherian VM, Hephzibah J, Prabhu AJ, Thomas N, Paul TV (2017) Musculoskeletal oncogenic osteomalacia-An experience from a single centre in South India. J Orthop 14:184-188

377 Schober HC, Kneitz C, Fieber F, Hesse K, Schroeder H (2017) Selective blood sampling for FGF-23 in tumor-induced osteomalacia. Endocrinology, Diabetes and Metabolism Case Reports 2017

378 Singh D, Chopra A, Ravina M, Kongara S, Bhatia E, Kumar N, Gupta S, Yadav S, Dabadghao P, Yadav R, Dube V, Kumar U, Dixit M, Gambhir S (2017) Oncogenic osteomalacia: Role of Ga-68 DOTANOC PET/CT scan in identifying the culprit lesion and its management. British Journal of Radiology 90

379 Tekin S, Tanrikulu S, Hacisahinogullari H, Koc S, Tuncer S, Bilgiç B, Uzum AK, Ferihan A, Refik T (2017) An unusual localization of tumor induced osteomalacia. Osteoporosis International 28:S595

380 Tella SH, Amalou H, Wood BJ, Chang R, Chen CC, Robinson C, Millwood M, Guthrie LC, Xu S, Levy E, Krishnasamy V, Gafni RI, Collins MT (2017) Multimodality Image-Guided Cryoablation for Inoperable Tumor-Induced Osteomalacia. Journal of Bone and Mineral Research 32:2248-2256

381 Tonello L, Gatti AP, Neto JD, Teixeira UF, Goldoni MB, Ott Fontes PR, Sampaio JA, Lima LMP, Waechter FL (2017) A case of oncogenic osteomalacia owing to inguinal tumor. Oxford Medical Case Reports 2017:202-205

382 Veldhuis-Vlug A, Rotman M, Bisschop P, Appelma-Dijkstra N (2017) Hypophosphatemic ostemalacia: A diagnosis often overlooked. Journal of Bone and Mineral Research 32:S121

383 Verma A, Tewari S, Kannaujia A (2017) Perioperative management of patients with severe hypophosphataemia secondary to oncogenic osteomalacia: Our experience and review of literature. Indian Journal of Anaesthesia 61:590-593

384 Wu W, Wang C, Ruan J, Chen F, Li N, Chen F (2017) A case report of phosphaturic mesenchymal tumor-induced osteomalacia. Medicine (United States) 96

385 Yu WJ, He JW, Fu WZ, Wang C, Zhang ZL (2017) Reports of 17 Chinese patients with tumor-induced osteomalacia. Journal of Bone and Mineral Metabolism 35:298-307

386 Zhu W, Ma Q, Bian Y, Zhuang Q, Xia Z, Jin J, Weng X (2017) Total hip/knee arthroplasty in the treatment of tumor-induced osteomalacia patients: More than 1 year follow-up. PLoS ONE 12

387 Zuo QY, Wang H, Li W, Niu XH, Huang YH, Chen J, You YH, Liu BY, Cui AM, Deng W (2017) Treatment and outcomes of tumor-induced osteomalacia associated with phosphaturic mesenchymal tumors: Retrospective review of 12 patients. BMC Musculoskeletal Disorders 18

388 박영창, 서준오, 양규현 (2017) Oncogenic Osteomalacia with Multiple Insufficiency Fractures - A Case Report. Journal of the Korean Fracture Society 30:146-150

389 Annamalai AK, Gill AJ, Shinto A, Sivakumar R, Singhi PK, Prabhu VA, Gopalakrishnan C, Chelian M, Sridhar S, Shanthi R, Puri V, Gururaj N, Srinivasan KG, Clifton-Bligh R, Gurnell M (2018) Oncogenic osteomalacia. QJM 111:421-422

390 Barta V, Lalkiya N, Phan H, Miller I, Sachdeva M (2018) Fibroblast growth factor 23-secreting pancreaticobiliary malignancy. Journal of Onco-Nephrology 2:21-23

391 Batra CM, Jain S, Uphadhya S, Singh VP (2018) Tumor induced hypophosphatemic Osteomalacia. Indian Journal of Endocrinology and Metabolism 22:S78

392 Berglund JA, Gafni RI, Wodajo F, Cowen EW, El-Maouche D, Chang R, Chen CC, Guthrie LC, Molinolo AA, Collins MT (2018) Tumor-induced osteomalacia in association with PTEN-negative Cowden syndrome. Osteoporosis International 29:993-997

393 Busquet F, Gahier-Penhoat M, Lescour V, Maugars Y, Guillot P (2018) Contribution of ultrasound to diagnosing a phosphaturic mesenchymal tumor. Joint Bone Spine 85:639-640

394 Cheng Z, Song S, Han Y, Zou S, Zhu X (2018) Elevated 68Ga-DOTATATE activity in IgG4-related lymphadenopathy. Clinical Nuclear Medicine 43:773-776

395 Colangelo L, Cipriani C, Pepe J, Corsi A, Sonato C, Follacchio G, Cilli M, Gianni W, Ferrone F, Moreschini O, Fitzpatrick LA, Minisola S (2018) A Challenging Case of Tumor-Induced Osteomalacia: Pathophysiological and Clinical Implications. Calcified Tissue International 103:465-468

396 Ding J, Hu G, Wang L, Li F, Huo L (2018) Increased Activity Due to Fractures Does Not Significantly Affect the Accuracy of68Ga-DOTATATE PET/CT in the Detection of Culprit Tumor in the Evaluation of Tumor-Induced Osteomalacia. Clinical Nuclear Medicine 43:880-886

397 Ding J, Wang L, Zhang S, Li F, Huo L (2018) Recurrent/residual intracranial phosphaturic mesenchymal tumor revealed on 68Ga-DOTATATE PET/CT. Clinical Nuclear Medicine 43:674-675

398 Doshi K, Spangehl M, Yang M (2018) Gallium-68 dodadate PET/CT scan for localization of tumorinduced osteomalacia. Endocrine Reviews 39

399 Dutta D, Pandey RK, Gogoi R, Solanki N, Madan R, Mondal A, Dogra S, Thapa P (2018) Occult phosphaturic mesenchymal tumour of femur cortex causing oncogenic osteomalacia - diagnostic challenges and clinical outcomes. Endokrynol Pol 69:205-210

400 Gou M, Ma Z (2018) Osteomalacia, renal Fanconi syndrome, and bone tumor. J Int Med Res 46:3487-3490

401 Ha S, Park S, Kim H, Go H, Lee SH, Choi JY, Hong JY, Ryu JS (2018) Successful Localization Using 68Ga-DOTATOC PET/CT of a Phosphaturic Mesenchymal Tumor Causing Osteomalacia in a Patient with Concurrent Follicular Lymphoma. Nuclear Medicine and Molecular Imaging 52:462-467

402 He Q, Xu Z, Zhang B, Hu W, Zhang X (2018) Tumor-Induced Osteomalacia Caused by a Parotid Basal Cell Adenoma Detected by 68Ga-DOTANOC PET/CT. Clinical Nuclear Medicine 43:e198-e199

403 Kane SV, Kakkar A, Oza N, Sridhar E, Pai PS (2018) Phosphaturic mesenchymal tumor of the nasal cavity and paranasal sinuses: A clinical curiosity presenting a diagnostic challenge. Auris Nasus Larynx 45:377-383

404 Li J, Huang Y, Yang F, Zhang Q, Chen D, Wang Q (2018) Sinonasal hemangiopericytoma caused hypophosphatemic osteomalacia: A case report. Medicine (United States) 97

405 Li L, Wang S-X, Wu H-M, Luo D-L, Dong G-F, Feng Y, Zhang X (2018) Acquired hypophosphatemic osteomalacia is easily misdiagnosed or neglected by rheumatologists: A report of 9 cases. Experimental and Therapeutic Medicine 15:5389-5393

406 Matani S, Barengolts E (2018) Cinacalcet as an adjuvant treatment and prevention of hypercalcemia after FGF23 tumor resection: two case reports of long-standing tumor induced osteomalacia (TIO). Endocrine Reviews 39

407 Muir C, Szajer J, Mansberg R, Russo R, Banh J, Boyle R, Crawford BA (2018) Low bone mineral density in a man with recurrent minimal trauma fractures: a cauTIOnary tale of unrecognised hypophosphataemia. Internal Medicine Journal 48:1003-1004

408 Mumford E, Marks J, Wagner T, Gallimore A, Gane S, Walsh SB (2018) Oncogenic osteomalacia: diagnosis, localisation, and cure. The Lancet Oncology 19:e365

409 Nakamura K, Ohishi M, Matsunobu T, Nakashima Y, Sakamoto A, Maekawa A, Oda Y, Iwamoto Y (2018) Tumor-induced osteomalacia caused by a massive phosphaturic mesenchymal tumor of the acetabulum: A case report. Modern Rheumatology 28:906-910

410 Paquet M, Gauthé M, Zhang Yin J, Nataf V, Bélissant O, Orcel P, Roux C, Talbot JN, Montravers F (2018) Diagnostic performance and impact on patient management of 68Ga-DOTA-TOC PET/CT for detecting osteomalacia-associated tumours. European Journal of Nuclear Medicine and Molecular Imaging 45:1710-1720

411 Ramsli T, Valeur J, Pretorius M, Gerlyng P (2018) Severe, reversible dysphagia and malnutrition in a patient with tumour-induced hypophosphataemia. BMJ Case Rep 2018

412 Sam JE, Kalra P (2018) Tumor induced osteomalacia. Indian Journal of Endocrinology and Metabolism 22:S86

413 Shi Z, Deng Y, Li X, Li Y, Cao D, Coossa VS (2018) CT and MR imaging features in phosphaturic mesenchymal tumor-mixed connective tissue: A case report. Oncology Letters 15:4970-4978

414 Spaleniak S, Kade G, Kidziński R, Mazurek A, Lubas A, Niemczyk S (2018) Tumor-induced hypophosphatemic osteomalacia as a rare cause of bone pain. Polish Archives of Internal Medicine 128:189-191

415 Wang L, Zhang S, Jing H, Chen L, Wang Z, Li F (2018) The Findings on Bone Scintigraphy in Patients with Suspected Tumor-Induced Osteomalacia Should Not Be Overlooked. Clinical Nuclear Medicine 43:239-245

416 Yamada Y, Kinoshita I, Kenichi K, Yamamoto H, Iwasaki T, Otsuka H, Yoshimoto M, Ishihara S, Toda Y, Kuma Y, Setsu N, Koga Y, Honda Y, Inoue T, Yanai H, Yamashita K, Ito I, Takahashi M, Ohga S, Furue M, Nakashima Y, Oda Y (2018) Histopathological and genetic review of phosphaturic mesenchymal tumours, mixed connective tissue variant. Histopathology 72:460-471

417 Yavropoulou MP, Poulios C, Foroulis C, Tournis S, Hytiroglou P, Kotsa K, Kessisoglou I, Zebekakis P (2018) Distant lung metastases caused by a histologically benign phosphaturic mesenchymal tumor. Endocrinology, Diabetes and Metabolism Case Reports 2018

418 Zhou Z, Yue H, Xu J, Xu X, Guo Q, Chen X, Zhang Z, Bao C (2018) A case with tumour-induced osteomalacia misdiagnosed as axial spondyloarthritis. Rheumatology (Oxford) 57:2242-2243

419 Acharya RP, Won AM, Moon BS, Flint JH, Roubaud MS, Williams MD, Hessel AC, Murphy WA, Chambers MS, Gagel RF (2019) Tumor-induced hypophosphatemic osteomalacia caused by a mesenchymal tumor of the mandible managed by a segmental mandibulectomy and microvascular reconstruction with a free fibula flap. Head and Neck 41:E93-E98

420 Adnan Z, Nikomarov D, Weiler-Sagie M, Maor NR (2019) Phosphaturic mesenchymal tumors among elderly patients: a case report and review of literature. Endocrinology Diabetes and Metabolism Case Reports

421 Agarwal N, Kale S, Kumari K (2019) Tumor-induced Osteomalacia due to a Phosphaturic Mesenchymal Tumor in the Cervical Spine: A Case Report and Literature Review. Neurology India 67:1334-1340

422 Aslam F, Chivers FS, Doshi KB, Chang-Miller A (2019) Positive HLA-B27 and sacroiliitis is not always spondyloarthritis. International Journal of Rheumatic Diseases 22:2213-2217

423 Bisceglia M, Galliani CA, Orcioni GF, Perrone E, Del Giudice A, Scillitani A (2019) Phosphaturic Mesenchymal Tumor of Soft Tissue of the Foot: Report of a Case With Review of the Literature. Advances in Anatomic Pathology 26:320-328

424 Hadelsberg UP, Doviner V, Frankel M, Gonen L, Munter G, Margalit N (2019) A rare brain tumor encountered: Phosphaturic mesenchymal tumor. Case report and review of the literature. Interdisciplinary Neurosurgery: Advanced Techniques and Case Management 16:91-94

425 Iftikhar B, Hussain F, Uzair M (2019) A CASE REPORT ON "TIO" (TUMOUR INDUCED OSTEOMALACIA) PRESENTING MUSCULAR WEAKNESS AND NON-SPECIFIC WEAKNESS. Indo American Journal of Pharmaceutical Sciences 6:1778-1781

426 Indirli R, Guabello G, Longhi M, Niada S, Maruca K, Mora S, Maggioni M, Corbetta S (2019) FGF23-related hypophosphatemia in patients with low bone mineral density and fragility fractures: challenges in diagnosis and management. Journal of Endocrinological Investigation

427 Jia C, Shao F, Yang M, Qin C, Lan X (2019) Giant Cell Tumor of Tendon Sheath Revealed on 68Ga-DOTA-TATE PET/CT in a Patient with Suspicious Tumor-Induced Osteomalacia. Clinical Nuclear Medicine 44:496-498

428 John JR, Hephzibah J, Oommen R, Shanthly N, Mathew D (2019) Ga-68 DOTATATE positron emission tomography-computed tomography imaging in oncogenic osteomalacia: Experience from a Tertiary Level Hospital in South India. Indian Journal of Nuclear Medicine 34:188-193

429 Kao YH, Li YC, Yeh LR, Tsai JW, Li JY, Tu YK, Shih SR (2019) Tumor-Induced Osteomalacia Treated as Ankylosing Spondylitis and Osteoporotic Compression Fracture. Journal of clinical rheumatology : practical reports on rheumatic & musculoskeletal diseases

430 Kaur T, Rush ET, Bhattacharya RK (2019) Phosphaturic Mesenchymal Heel Tumor Presenting with Tumor-Induced Osteomalacia. AACE Clin Case Rep 5:e138-e141

431 Kerr A, Rimmer R, Rosen MR, Evans JJ, Tuluc M, Mardekian SK (2019) Phosphaturic mesenchymal tumor of the nasal cavity: Clinicopathologic correlation is essential for diagnosis. Human Pathology: Case Reports 15:33-36

432 Kinoshita Y, Takashi Y, Ito N, Ikegawa S, Mano H, Ushiku T, Fukayama M, Nangaku M, Fukumoto S (2019) Ectopic expression of Klotho in fibroblast growth factor 23 (FGF23)-producing tumors that cause tumor-induced rickets/osteomalacia (TIO). Bone Reports 10

433 Krishnappa B, Jadhav SR, Lila AR, Bandgar TR (2019) Tumour-induced osteomalacia due to an intra-abdominal mesenchymal tumour. BMJ Case Reports 12

434 Kurien R, Rupa V, Thomas M (2019) Varied presentation of sinonasal phosphaturic mesenchymal tumour: report of a case series with follow-up. European Archives of Oto-Rhino-Laryngology

435 Layman AAK, Joshi S, Shah S (2019) Metastatic prostate cancer presenting as tumour-induced osteomalacia. BMJ Case Reports 12

436 Li Y, Li Y, Hui M, Liu Y, Liu X, Jin J, Gao P (2019) Comparison of surgical treatments of tumor-induced osteomalacia in different locations in the lower limbs: A retrospective study. Medicine 98:e14846

437 Liu S, Zhou X, Song A, Huo Z, Wang Y, Xia W, Liu Y (2019) Successful treatment of tumor-induced osteomalacia causing by phosphaturic mesenchymal tumor of the foot. Medicine 98:e16296

438 Mishra SK, Kuchay MS, Sen IB, Garg A, Baijal SS, Mithal A (2019) Successful Management Of Tumor-Induced Osteomalacia with Radiofrequency Ablation: A Case Series. JBMR Plus 3

439 Mishra T, Desouza MA, Patel K, Mazumdar GA (2019) Phosphaturic Mesenchymal Tumors Involving Skull Bones: Report of Two Rare Cases. Asian J Neurosurg 14:253-255

440 Pal R, Bhadada SK, Singhare A, Bhansali A, Kamalanathan S, Chadha M, Chauhan P, Sood A, Dhiman V, Sharma DC, Saikia UN, Chatterjee D, Agashe V (2019) Tumor-induced osteomalacia: Experience from three tertiary care centers in India. Endocrine Connections 8:266-276

441 Panciera DT, Murè C, Piazza A, D'Aco LF, Aresi G, Cagnoni L (2019) Tumour-induced osteomalacia: A case report of craniofacial localization. Otolaryngology Case Reports 10:27-31

442 Paul J, Cherian KE, Kapoor N, Paul TV (2019) Treating osteoporosis: A near miss in an unusual case of FGF-23 mediated bone loss. BMJ Case Reports 12

443 Qian Y, Dai Z, Zhu C, Ruan L, Thapa S, Wu C (2019) Tumor-induced osteomalacia with the culprit lesion located in the palm: a case report. Journal of International Medical Research 47:2240-2247

444 Richardson AL, Richardson OK (2019) Phosphaturic mesenchymal tumor: Case report. Radiology Case Reports 14:1518-1524

445 Rodionova SS, Snetkov AI, Akinshina AD, Bulycheva IV, Torgashin AN, Grebennikova TA, Belaya ZE, Agafonova EM, Toroptsova NV, Nikitinskaya OА (2019) Hypophosphatemic osteomalacia induced by FGF23-secreting tumor of the left femur. Nauchno-Prakticheskaya Revmatologiya 57:708-712

446 Roxas MCAR, Sandoval MA (2019) Hypophosphatemia and metabolic bone disease: A rare case of tumor induced osteomalacia. Osteoporosis and Sarcopenia 5:S14

447 Savva C, Adhikaree J, Madhusudan S, Chokkalingam K (2019) Oncogenic osteomalacia and metastatic breast cancer: a case report and review of the literature. Journal of Diabetes and Metabolic Disorders 18:267-272

448 Seemann L, Padala SA, Mohammed A, Belayneh N (2019) Tumor-Induced Osteomalacia and the Importance of Plasma Fibroblast Growth Factor 23 as an Indicator: Diagnostic Delay Leads to a Suicide Attempt. Journal of Investigative Medicine High Impact Case Reports 7

449 Shah R, Lila AR, Ramteke-Jadhav S, Patil V, Mahajan A, Sonawane S, Thadani P, Dcruz A, Pai P, Bal M, Kane S, Shah N, Bandgar T (2019) Tumor induced osteomalacia in head and neck region: Single center experience and systematic review. Endocrine Connections 8:1330-1353

450 Shenbaghavalli T, Harshavardhan JKG, Menon PG (2019) A Rare Case of Phosphaturic Tumor/Oncogenic Osteomalacia - Diagnostic Challenges and Management Algorithm. J Orthop Case Rep 9:49-52

451 Shi X, Jing H, Li F, Zhao Y, Wang Z, Huo L (2019) 99mTc-HYNIC-TOC in the Evaluation of Recurrent Tumor-Induced Osteomalacia. Clinical Nuclear Medicine 44:209-213

452 Soumya SL, Cherian KE, Gupta RD, Poonnoose PM, Hephzibah J, Prabhu AJ, Paul TV, Kapoor N (2019) An uncommon cause of polyarthralgia. J Family Med Prim Care 8:1801-1803

453 Tang D, Wang X, Zhang Y, Mi XX (2019) Oncogenic osteomalacia caused by a phosphaturic mesenchymal tumor of the femur: A case report. World Journal of Clinical Cases 7:2081-2086

454 Wang X, Gao J, Han S, Li Y (2019) Spinal phosphaturic mesenchymal tumors: Case report and literature review. Journal of Clinical Neuroscience 63:234-239

455 Wei H, Liu R, Wang ZH, Yao ZQ (2019) [Hypophosphatemic osteomalacia caused by urinary mesenchymal tumor: A case report]. Beijing Da Xue Xue Bao Yi Xue Ban 51:1169-1172

456 Abi-Ghanem AS, Chouairy CJ, Meguerian Z, Azar L (2020) A 49-Year-Old Man With Debilitating Aches and Pains and a Mysterious Culprit. Arthritis Care and Research 72:1-8

457 Bhalla MI, Wirtz KM, Fair ES, Bucklan DJ (2020) (68)Ga-DOTATATE positron emission tomography/computed tomography to detect the recurrence of phosphaturic mesenhcymal tumor-induced osteomalacia. World J Nucl Med 19:78-81

458 Colazo JM, Thompson RC, Covington NV, Dahir KM (2020) An intracranial mass causing tumor-induced osteomalacia (TIO): Rapid and complete resolution of severe osteoporosis after surgical resection. Radiology Case Reports 15:492-497

459 Day AL, Gutiérrez OM, Guthrie BL, Saag KG (2020) Burosumab in tumor-induced osteomalacia: A case report. Joint Bone Spine 87:81-83

460 Kawthalkar AS, Janu AK, Deshpande MS, Gala KB, Gulia A, Puri A (2020) Phosphaturic Mesenchymal Tumors from Head to Toe: Imaging Findings and Role of the Radiologist in Diagnosing Tumor-Induced Osteomalacia. Indian J Orthop 54:215-223

461 Liu S, Zhou X, Song A, Huo Z, Wang Y, Liu Y (2020) Surgical treatment of recurrent spinal phosphaturic mesenchymal tumor-induced osteomalacia: A case report. Medicine (United States) 99

462 Long Y, Shao F, Lan X (2020) Mediastinal Epithelioid Hemangioendothelioma Revealed on 68Ga-DOTATATE PET/CT. Clinical nuclear medicine 45:414-416

463 Muro Bushart N, Tharun L, Oheim R, Paech A, Kiene J (2020) [Tumor-induced osteomalacia caused by an FGF23-secreting myopericytoma : Case report and literature review]

Tumorinduzierte Osteomalazie, verursacht durch ein FGF23-sezernierendes Myoperizytom : Literaturubersicht und Fallbericht. Orthopade 49:1-9

464 Oyama N, Kojima-Ishii K, Toda N, Matsuo T, Tocan V, Ohkubo K, Oba U, Koga Y, Setsu N, Yamada Y, Kohashi K, Nakashima Y, Oda Y, Ihara K, Ohga S (2020) Malignant transformation of phosphaturic mesenchymal tumor: a case report and literature review. Clin Pediatr Endocrinol 29:69-75

465 Pal R, Agrawal K, Gupta S, Bhansali A, Behera A, Bhadada SK (2020) Worsening of unrecognized tumour-induced osteomalacia with inadvertent use of recombinant human parathyroid hormone. Endokrynologia Polska 71:102-103

466 Srinivasan M, Ross R, Abramson M (2020) RENAL PHOSPHATE WASTING DUE TO TUMOR-INDUCED OSTEOMALACIA IN A BREAST CANCER PATIENT. American Journal of Kidney Diseases 75:639-640

467 Then C, Asbach E, Bartsch H, Thon N, Betz C, Reincke M, Schmidmaier R (2020) Fibroblast Growth Factor 23-Producing Phosphaturic Mesenchymal Tumor with Extraordinary Morphology Causing Oncogenic Osteomalacia. Medicina (Kaunas, Lithuania) 56

468 Wu N, Zhang Z, Zhou X, Zhao H, Ming Y, Wu X, Zhang X, Yang XZ, Zhou M, Bao H, Chen W, Wu Y, Liu S, Wang H, Niu Y, Li Y, Zheng Y, Shao Y, Gao N, Yang Y, Liu Y, Li W, Liu J, Zhang N, Yang X, Xu Y, Li M, Sun Y, Su J, Zhang J, Xia W, Qiu G, Liu Y, Liu J, Wu Z (2020) Mutational landscape and genetic signatures of cell-free DNA in tumour-induced osteomalacia. Journal of Cellular and Molecular Medicine 24:4931-4943
